# Elucidating the Role of Reduction Kinetics in the Phase-Controlled Growth on Preformed Nanocrystal Seeds: A Case Study of Ru

**DOI:** 10.1021/jacs.4c01725

**Published:** 2024-03-30

**Authors:** Quynh N. Nguyen, Eun Mi Kim, Yong Ding, Annemieke Janssen, Chenxiao Wang, Kei Kwan Li, Junseok Kim, Kristen A. Fichthorn, Younan Xia

**Affiliations:** † School of Chemistry and Biochemistry, Georgia Institute of Technology, Atlanta, Georgia 30332, United States; ‡ Department of Chemical Engineering, The Pennsylvania State University, University Park, Pennsylvania 16803, United States; § School of Materials Science and Engineering, Georgia Institute of Technology, Atlanta, Georgia 30332, United States; ⊥ The Wallace H. Coulter Department of Biomedical Engineering, Georgia Institute of Technology and Emory University, Atlanta, Georgia 30332, United States

## Abstract

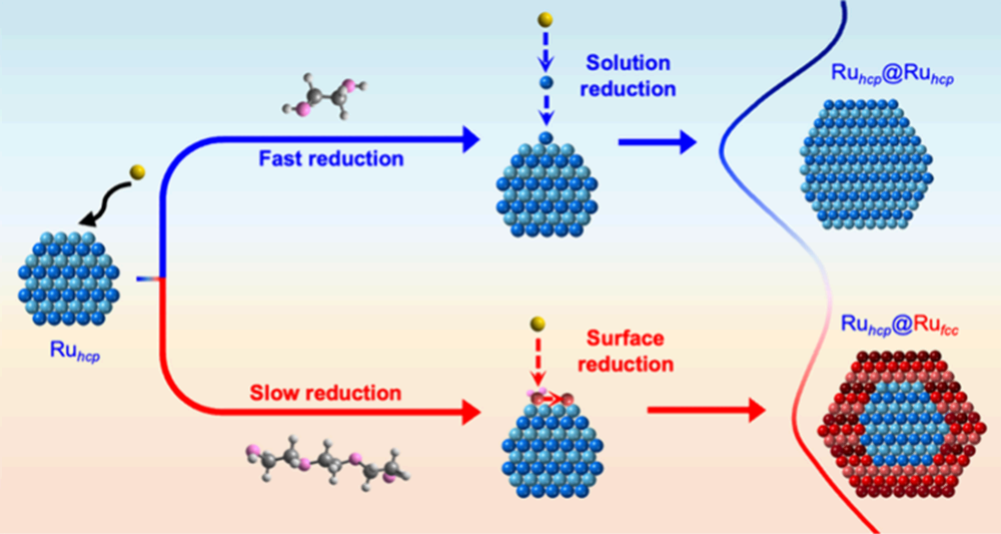

This study demonstrates the crucial role of reduction kinetics in phase-controlled synthesis of noble-metal nanocrystals using Ru nanocrystals as a case study. We found that the reduction kinetics played a more important role than the templating effect from the preformed seed in dictating the crystal structure of the deposited overlayers despite their intertwined effects on successful epitaxial growth. By employing two different polyols, a series of Ru nanocrystals with tunable sizes of 3–7 nm and distinct patterns of crystal phase were synthesized by incorporating different types of Ru seeds. Notably, the use of ethylene glycol and triethylene glycol consistently resulted in the formation of Ru shell in natural hexagonal close-packed (*hcp*) and metastable face-centered cubic (*fcc*) phases, respectively, regardless of the size and phase of the seed. Quantitative measurements and theoretical calculations suggested that this trend was a manifestation of the different reduction kinetics associated with the precursor and the chosen polyol, which, in turn, affected the reduction pathway (solution versus surface) and packing sequence of the deposited Ru atoms. This work not only underscores the essential role of reduction kinetics in controlling the packing of atoms and thus the phase taken by Ru nanocrystals but also suggests a potential extension to other noble-metal systems.

## Introduction

The properties of noble-metal nanocrystals for specific applications have been traditionally tuned by tailoring parameters such as size, shape, and internal structure.^1−4^ Exploring the polymorphism of nanocrystals, specifically controlling their crystal structures or phases, represents a nascent research area with many mechanistic details yet to be elucidated, reminiscent of the early days of shape-controlled synthesis.^5−8^ While shape control only involves surface atoms or a few internal atoms to generate twin boundaries and stacking faults, the modulation of crystal structure requires the rearrangement of essentially all atoms in order to form different phases.^7^ In recent years, seed-mediated growth has been established as a simple and versatile method for achieving phase-controlled synthesis.^9−13^ Under optimal conditions, the deposited shell can replicate both the crystal and surface structures of the preformed seed, leading to the formation of a metastable phase. With the ability to simultaneously maneuver thermodynamic and kinetic factors in a colloidal synthesis, such phase-controlled growth allows for good reproducibility between batches, tight controls over the size and shape, and a systematic examination of parameters responsible for phase evolution.^14,15^ Although many studies have delved into the effects of overlayer thickness, template shape (surface structure), and size (bulk versus surface energy) on phase evolution,^16−20^ creating nanocrystals in a metastable phase of more than six atomic layers in thickness via epitaxial growth still presents a major challenge. The explicit role(s) played by reaction kinetics in affecting the packing of atoms in the bulk is yet to be resolved.

Unlike other noble metals, such as Ag, Au, Pd, and Pt, research on the colloidal synthesis of Ru nanocrystals is relatively scarce. Most studies have focused on their thermodynamically stable hexagonal close-packed (*hcp*) phase and the metastable face-centered cubic (*fcc*) phase.^7^ The difference between these phases lies in the arrangement of the third atomic layer (ABCABC for *fcc versus* ABABAB for *hcp*), a subtle variation that significantly influences catalytic performance by altering interactions between reaction intermediates and surface atoms.^16,20−23^ As reported in the literature, various factors can impact the packing of Ru atoms. For the most robust polyol synthesis involving homogeneous nucleation, chemical selection was crucial for determining the crystal phase. The first report of *fcc*-Ru nanocrystals utilized polyol reduction with an appropriate pair of precursor and reductant.^24^ Specifically, Ru nanocrystals derived from RuCl_3_ and ethylene glycol (EG) adopted the conventional *hcp* phase, while those from Ru(acac)_3_ and triethylene glycol (TEG) favored the *fcc* phase. It was hypothesized that the stabilization of Ru(III) ions by the acac^–^ ligand decelerated the reduction kinetics, causing atypical atomic packing.^25^ This trend was also attributed to the similarity in distance between the oxygen atoms in acac^–^ and the Ru atoms on *fcc* facets, suggesting preferential ligand-*fcc* facet binding to facilitate their formation. However, one study also successfully prepared *fcc*-Ru nanocrystals in TEG and *hcp*-Ru nanocrystals in EG using either RuCl_3_ or Ru(acac)_3_ as the precursor.^26^ This finding highlights the influence of the chemical species on phase control; yet, it is debatable whether this effect was actually exerted through the reduction kinetics. A recent study shed light on this issue by elucidating a quantitative correlation between the crystal phase of seeds formed during nucleation and the initial reduction rate of the precursor.^27^ While the authors were able to obtain Ru nanocrystals with varying percentages of the metastable *fcc* phase, decoupling the effect of reaction kinetics from the nucleation process would lead to a more precise control over the crystal phase and thus enhance the purity of the products.

With regard to the seed-mediated growth, prior studies have mainly harnessed the templating effect from the seeds to produce Ru-based nanocrystals featuring *fcc* structure and well-controlled shapes.^16−20,28−30^ It is still unclear if the reduction kinetics, as controlled by the type or concentration of the precursor and reducing agent, reaction temperature, and injection rate, also affect the nucleation and growth modes, ultimately determining the packing of Ru atoms. Some compelling evidence can be found in the deposition of Ru atoms on Pd nanocubes.^17,20^ When the amount of Ru precursor injected into the reaction was increased, the deposition of Ru atoms was switched from layer-by-layer to layer-plus-island and further to island mode as a result of faster reduction kinetics and thus self-nucleation. In conjunction with the variation in growth mode, the packing of Ru atoms was changed accordingly from *fcc*, through a mix of *fcc* and *hcp*, to *hcp*. This observation accentuates how the reduction kinetics of the precursor, even in the presence of the templating effect from seeds, can drastically alter the packing of Ru atoms.

The intricate roles played by reaction kinetics in phase-selective epitaxial growth have also been manifested in other noble metals and II–IV semiconductor systems. For instance, utilizing *fcc*-Au nanospheres as seeds enabled the formation of metastable *hcp*-Au hexagonal stars when involving ethylenediaminetetraacetic acid to complex with Au(III) and 2-phospho-l-ascorbic acid trisodium salt (Asc-2P) as a reducing agent to maneuver the reduction kinetics.^31^ The difference in atomic arrangement was linked to the interaction between the phosphate groups of Asc-2P and Au atoms. It should be pointed out that certain chemical species can not only affect the reduction kinetics but also bind to the metal surface to dictate atomic packing and thus the crystal phase of the resultant nanocrystals.^32−35^ In semiconductor nanocrystals, cadmium phosphonate with a long hydrocarbon chain was found to exclusively promote the formation of wurtzite phase, irrespective of whether the initial CdSe seeds took zinc blend or wurtzite phase.^36^ This result highlighted the predominant influence of the chemical environment on regulating the phase of CdSe nanocrystals. Nevertheless, no kinetic measurement was conducted, while the explicit mechanism of ligand-surface coordination remains elusive, necessitating additional studies for a conclusive interpretation. All of these findings collectively attest that a successful phase-controlled synthesis of metal nanocrystals may rely on the synergy arising from the templating effect and the chemical environment due to their specific impacts on reduction kinetics and ligand-surface interactions.

Intrigued by varied outcomes in prior syntheses, even with the use of seeds in desired phases, this study aims to discern which factor—the reduction kinetics or the templating effect from the seed, exerts a greater impact on dictating the crystal phase of the deposited overlayers. With Ru as a model system, we demonstrated a correlation between the phase and the initial reduction rate and revealed the explicit role played by reduction kinetics in phase-controlled growth on preformed seeds. By leveraging polyols with distinct reducing powers and Ru nanocrystals of varying phases as templates, a series of Ru nanocrystals with tunable sizes of 3–7 nm and different patterns of crystal phases were synthesized. Our findings suggest that the reduction kinetics played a more dominant role than the templating effect of the seeds in dictating the crystal phase of the deposited Ru, despite their intertwined effects on successful epitaxial growth. Specifically, EG consistently favored the formation of *hcp*-Ru overlayers, while TEG led to metastable *fcc*-Ru, regardless of the size and crystal phase of the preformed seeds. Based on the findings in Density Functional Theory (DFT) total energy calculations, this trend was attributed to the different reduction kinetics of the precursor in the respective polyol, influencing the reduction pathway (solution versus surface reduction) and thereby the packing of Ru atoms. Explicating the critical role of the chemical environment, this work highlights the intricate balance between the templating effect and reaction kinetics in generating the desired phase during nanocrystal growth.

## Results and Discussion

### Selection and Rationale of a Model System

The selection of monometallic Ru nanocrystals as a model system in this study has several merits. First, Ru is one of the few noble metals that can be prepared as nanocrystals purely in *hcp* or *fcc* phase by altering the polyol employed in a one-pot synthesis^24,26^ or by leveraging seeds with an intrinsic *fcc* structure.^28−30^ These robust protocols offer a framework to fine-tune specific parameters to attain Ru nanocrystals in the specific phases, establishing a well-controlled platform to systematically probe the influence of reduction kinetics. Second, employing a monometallic system enables us to exclusively attribute the observed variations in phase to the reduction kinetics, avoiding complications introduced by the lattice mismatch intrinsic to a bimetallic system. As a factor inherently encoded in the seed, a large lattice mismatch can force the deposited shell to adopt the native phase and thus minimize the total surface energy, while the templating effect from the seed tends to prevail over other factors when the lattice mismatch is minimal.^9,37,38^ For instance, an *fcc-*Ru shell could be generated in a synthesis based on EG and *fcc*-Pd nanocrystal seeds due to the prominent templating effect stemming from the small lattice mismatch between Pd and *fcc*-Ru (1.8%, 3.89 versus 3.82 Å), when coupled with the symmetry alignment across different facets for the balance of surface and bulk energies.^18−20^ However, the templating effect in the Pd–Ru system would gradually vanish when the Ru shell was beyond *ca*. 5 atomic layers,^16,17^ possibly due to the dominance of other effects (*i.e*., growth kinetics) in dictating phase evolution. Third, incorporating a second metal inevitably introduces impurities into the Ru shell due to interdiffusion,^16,29^ potentially altering the *fcc* crystallization of Ru and thus overshadowing the effects from other factors or parameters.

**Figure 1:**
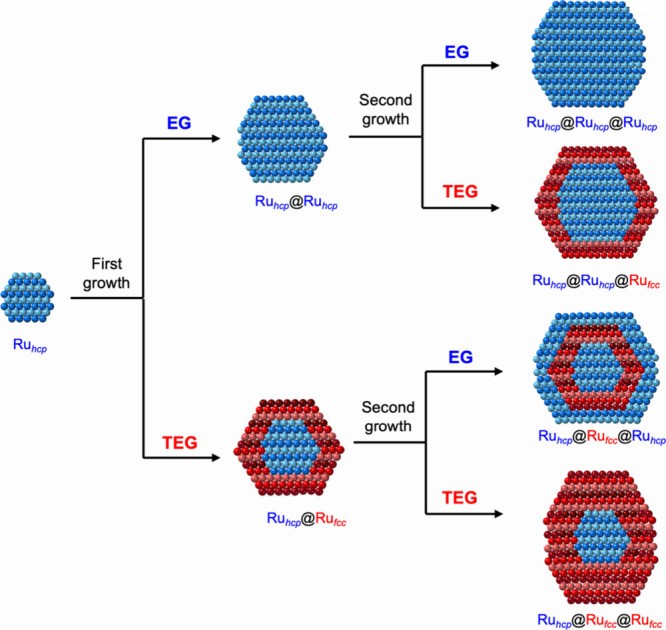
Schematic illustration of four synthetic routes to the preparation of Ru nanocrystals with different patterns of phases by simply switching the polyol used in the overgrowth process.

Although many compounds have been documented as reducing agents and/or solvents for Ru nanocrystal synthesis,^21,39−41^ we opted for polyols, specifically EG and TEG. They stand out among other candidates due to their dual functionalities in a colloidal synthesis, systematic variation in molecular structure, and different reduction potentials to enable distinct reduction kinetics.^42^ Overall, all of the experiments in this study involved the reduction of Ru(acac)_3_ by EG or TEG at 180 °C in the presence of poly(vinylpyrrolidone) (PVP) as a colloidal stabilizer. No capping agent was added to ensure direct interactions between the polyol molecules and nanocrystal surface while avoiding its kinetic and thermodynamic alterations to the evolution of crystal phase. Figure 1 shows a summary of the two rounds of epitaxial overgrowth involved in our study, distinguished solely by the employment of EG or TEG to reduce the precursor.

### Synthesis and Characterizations of the *hcp*-Ru Seeds

The synthesis started with the preparation of 3.1 nm Ru_*hcp*_ nanocrystals using a slightly modified protocol from the literature that involves the reduction of Ru(acac)_3_ by EG at 180 °C in the presence of PVP.^24^Figure S1A shows a typical transmission electron microscopy (TEM) image of the as-obtained nanocrystals, which had an average size of 3.1 ± 0.5 nm and a quasi-spherical shape. The phase of the product was characterized by X-ray diffraction (XRD) and high-resolution transmission electron microscopy (HRTEM) to ensure that any structural transformation or preservation in the next step(s) could be attributed to experimental conditions rather than the inherent characteristics of the seeds. The XRD pattern shows a broad peak at 2θ of 42.1° due to an overlap of the diffractions from (101̅0), (0002), and (101̅1) planes of *hcp*-Ru (Figure S1B). The overlapping XRD signal is largely attributed to the broadening of the three distinctive peaks of *hcp*-Ru in the region of 38–45° as a result of small crystallite size of the nanocrystals.^24,26,43^ The HRTEM image from an individual Ru nanocrystal confirmed the characteristic lattice spacings of *hcp*-Ru (Figure S1C), which is in agreement with the XRD pattern. Meanwhile, the corresponding fast Fourier transform (FFT) pattern also supported an *hcp* structure viewed along the [12̅13̅] zone axis (Figure S1D, E). The as-obtained *hcp*-Ru nanocrystals (denoted as Ru_*hcp*_ seeds thereafter) were directly used for the first round of overgrowth in EG without additional treatment. For subsequent procedures involving TEG, thorough washing with a mixture of acetone and ethanol was carried out to ensure the complete removal of residual EG and other impurities, mitigating the influence of undesired chemicals in the subsequent phase evolution.

### Growth of Ru Overlayers on Ru Seeds in Different Polyols

The first round of overgrowth was initiated by introducing the Ru(acac)_3_ precursor solution into a suspension of the 3.1 nm Ru_*hcp*_ seeds in EG or TEG to initiate the overgrowth of Ru. Depending on the experimental conditions, the deposition of Ru can proceed via two pathways in the presence of seeds: epitaxial layer-by-layer growth through heterogeneous nucleation and island/attachment growth through homogeneous (or self-) nucleation.^14,44^ For the former, Ru atoms are generated via solution or surface reduction, followed by their heterogeneous nucleation and deposition on the seed to produce a smooth Ru shell.^45−47^ Conversely, Ru atoms are produced in the solution, followed by homogeneous nucleation of small nuclei, which then aggregate or attach to the seed.^2^ In this case, the final product usually comprises two distinct populations, one of which contains Ru particles with significantly different morphologies or much smaller sizes than the original seeds. Since the polyol has been found to influence the phase evolution of Ru during homogeneous nucleation,^24−26^ the occurrence of both nucleation events would overshadow the templating effect, complicating the differentiation of the individual contribution from solvent versus template in a seed-mediated synthesis. To discern their synergic or predominant roles in inducing the observed changes in the crystal phase, it is vital to ensure the dominance of heterogeneous nucleation and subsequent layer-by-layer growth.

**Figure 2:**
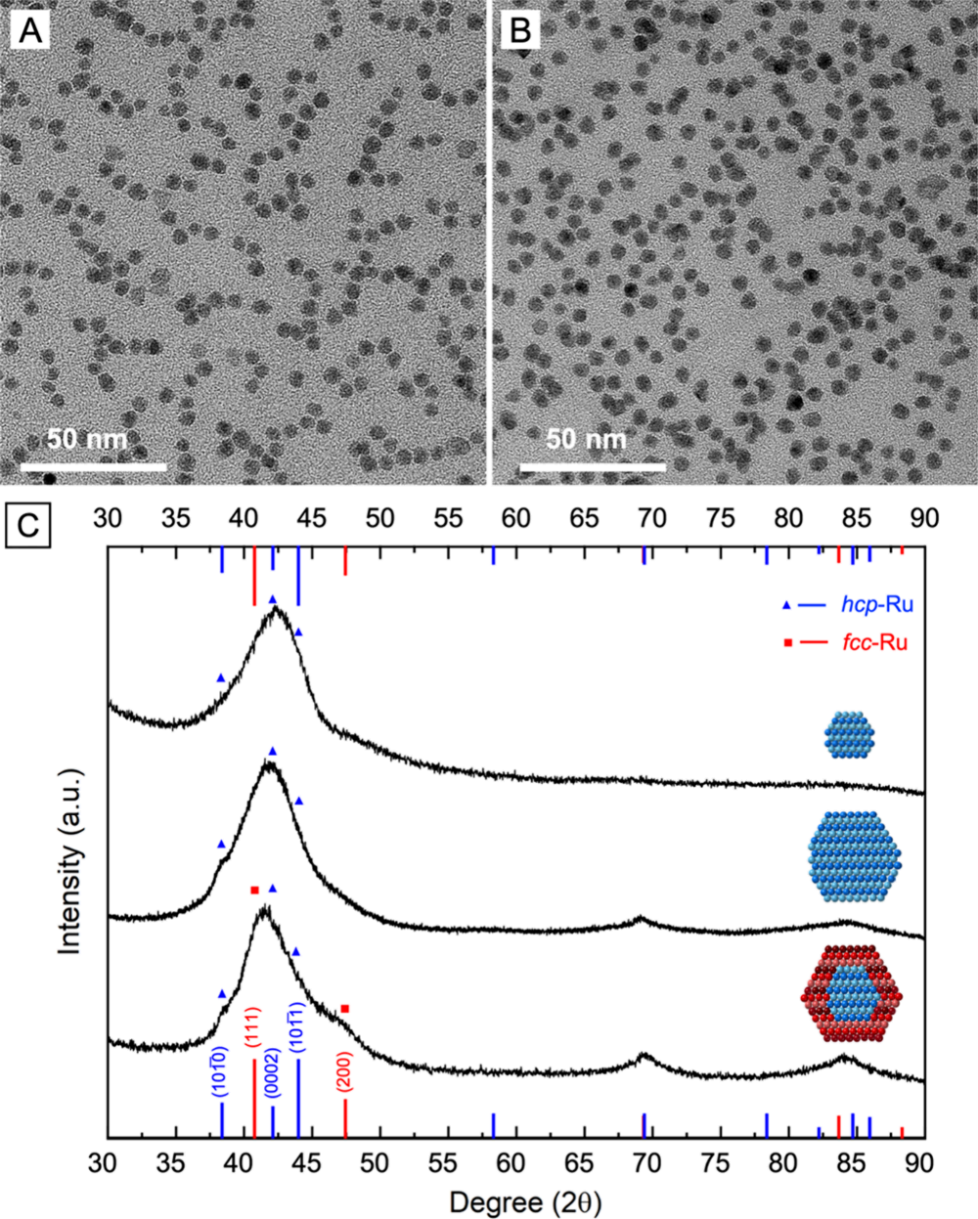
Ru@Ru nanocrystals with an *hcp* or *fcc* phase in the shell obtained through the first round of overgrowth. (A, B) TEM images of the Ru@Ru nanocrystals synthesized from the Ru_*hcp*_ seeds using (A) EG and (B) TEG, respectively. (C) XRD patterns of Ru_*hcp*_@Ru_*hcp*_ and Ru_*hcp*_@Ru_*fcc*_ nanocrystals produced in EG and TEG, respectively, together with that of the Ru_*hcp*_ seeds for comparison. The insets show the corresponding atomic model of the nanocrystals in a cross-sectional view. The red and blue lines correspond to the characteristic peaks of *fcc*-Ru (JCPDS No. 01–088–2333) and *hcp*-Ru (JCPDS No. 06–0663), respectively.

In principal, successful synthesis of nanocrystals in the phase of the template relies on the achievement of slow reduction kinetics and a faster atomic diffusion rate compared to the deposition rate to facilitate heterogeneous nucleation and layer-by-layer growth, respectively.^15,48^ In contrast, both homogeneous nucleation and island growth mode usually favor the formation of the thermodynamically stable phase.^20,49^ We examined various experimental parameters that could affect these kinetic factors, including the injection rate, reaction temperature, and speciation of the precursor. In the case of EG, we found that a relatively slow injection rate of 0.5 mL h^–1^, a high temperature of 180 °C, and the use of halide-free precursor offered an optimal combination of conditions to simultaneously achieve these goals. First, the precursor had to be introduced at a sufficiently slow rate to ensure a low and stable concentration of Ru atoms in the reaction mixture, thereby avoiding supersaturation and subsequent homogeneous nucleation.^48,50^ With a reduced supply of atoms, a slow injection rate directly decelerated the atom deposition rate, promoting layer-by-layer growth. When the injection rate was carefully controlled at a relatively slow rate of 0.5 mL h^–1^ using a syringe pump, Ru nanocrystals with a well-retained quasi-spherical shape and enlarged sizes were obtained (Figure 2A). No second population of particles with a much smaller size was observed, confirming the dominance of heterogeneous nucleation and uniform growth on the surface of the 3.1 nm Ru_*hcp*_ seeds to produce a smooth Ru shell. In comparison, If the injection rate was increased to 4 mL h^–1^, the surface of each Ru seed became very rough and was covered by a high density of small bumps, indicating the prevalence of homogeneous nucleation and island growth (Figure S2A). In this case, due to a higher concentration and thus faster reduction of the precursor, Ru atoms would self-nucleate in the solution and then aggregate into small particles, which subsequently attached to the surface of Ru seeds, driven by a desire to minimize the total surface energy.

The reaction temperature should also be taken into consideration since it affects not only the surface diffusion rate of adatoms but also the reduction kinetics, which will in turn influence the deposition rate of the Ru atoms.^14,20^ When the reaction was conducted at a higher temperature of 200 °C, homogeneous nucleation was triggered as the concentration of the newly formed Ru atoms was elevated while island growth was initiated as a result of the fast deposition rate, producing Ru nanocrystals with a high density of branched arms due to the attachment of small particles (Figure S2B). In contrast, an optimal temperature of 180 °C led to products with uniform size and smooth shell since both deposition and diffusion rates of the newly generated Ru atoms were adequate (Figure 2A). Further reducing the temperature to 160 °C caused no significant change in size to the final products because of the weakened reducing power of EG at this temperature and, thus, insufficient reduction of the precursor to ensure an adequate supply of Ru atoms (Figure S2C). Regarding the type of precursor, Ru(acac)_3_ was used to produce Ru atoms while avoiding possible oxidative etching and preferential capping to *fcc* facets commonly associated with halide ions.^8,49,51^ Moreover, the strong coordination of acac^–^ to Ru(III) ions significantly slowed down the reduction kinetics to help promote heterogeneous nucleation for the epitaxial growth of Ru.^25^ Indeed, when RuCl_3_ was used as a precursor, we obtained a polydisperse sample with the formation of tiny Ru particles in addition to a Ru shell due to the fast reduction rate and thus self-nucleation (Figure S2D). Taken together, all of the reaction parameters must be optimized to promote heterogeneous nucleation and layer-by-layer growth for the generation of a smooth, conformal shell while suppressing the formation of small Ru particles with phases determined solely by the polyol.

Figure 2A, B shows typical TEM images of the Ru nanocrystals obtained from the first round of overgrowth on the 3.1 nm Ru_*hcp*_ seeds using the standard protocol based on EG and TEG, respectively. Both products exhibited a relatively uniform size distribution, with average diameters of 5.0 ± 0.7 nm and 5.1 ± 1.2 nm, respectively. The increase in size from *ca*. 3 to 5 nm confirmed the successful deposition of Ru overlayers on the seeds. The crystal phase taken by the deposited Ru shell was determined by analyzing the XRD pattern of the obtained sample (Figure 2C). The XRD pattern of the sample produced in EG was similar to that of the Ru_*hcp*_ seeds, except for better separation of the three characteristic peaks of *hcp*-Ru in the region of 38–45° due to the increase in crystallite size. However, for the sample produced in TEG, the position of the main XRD peak shifted to 41.4°, between the reference peaks of *fcc*-Ru(111) and *hcp*-Ru(0002). Additionally, a broad shoulder peak appeared at 2θ of 47.4°. This peak could be assigned to the diffraction from the (200) planes of *fcc*-Ru, suggesting the existence of the *fcc* phase in the particles. The weak intensity of the shoulder peak could be attributed to the relatively small contribution from the *fcc* phase as a result of the thin *fcc*-Ru shell over the *hcp*-Ru core. From the XRD patterns, it can be concluded that even in the presence of a templating effect from the Ru_*hcp*_ seeds, the Ru shell adopted the native *hcp* phase when synthesized in EG while TEG favored the formation of metastable *fcc*-Ru shell, leading to the formation of homophased Ru_*hcp*_@Ru_*hcp*_ and heterophased Ru_*hcp*_@Ru_*fcc*_ nanocrystals, respectively.

One might argue that the crystal structure of the template could have been altered due to the interactions with TEG during the reaction, which might have led to the observed phase changes in the Ru shell. To address this issue, a control experiment was carried out, wherein the Ru_*hcp*_ seeds were mixed with PVP and heated in TEG at 180 °C for 22 h without introducing the Ru(III) precursor. From the TEM image and XRD pattern (Figure S3), we observed no changes to the size, morphology, or phase, reinforcing the argument that TEG influenced the deposition of Ru in a phase different from that of the seed rather than causing a phase change to the seed itself. This observation is consistent with the result from DFT calculation in that EG or TEG adsorption on the surface did not change the arrangement of Ru adatoms on the *hcp*-Ru(0001) template, which will be further discussed in the computational section.

**Figure 3:**
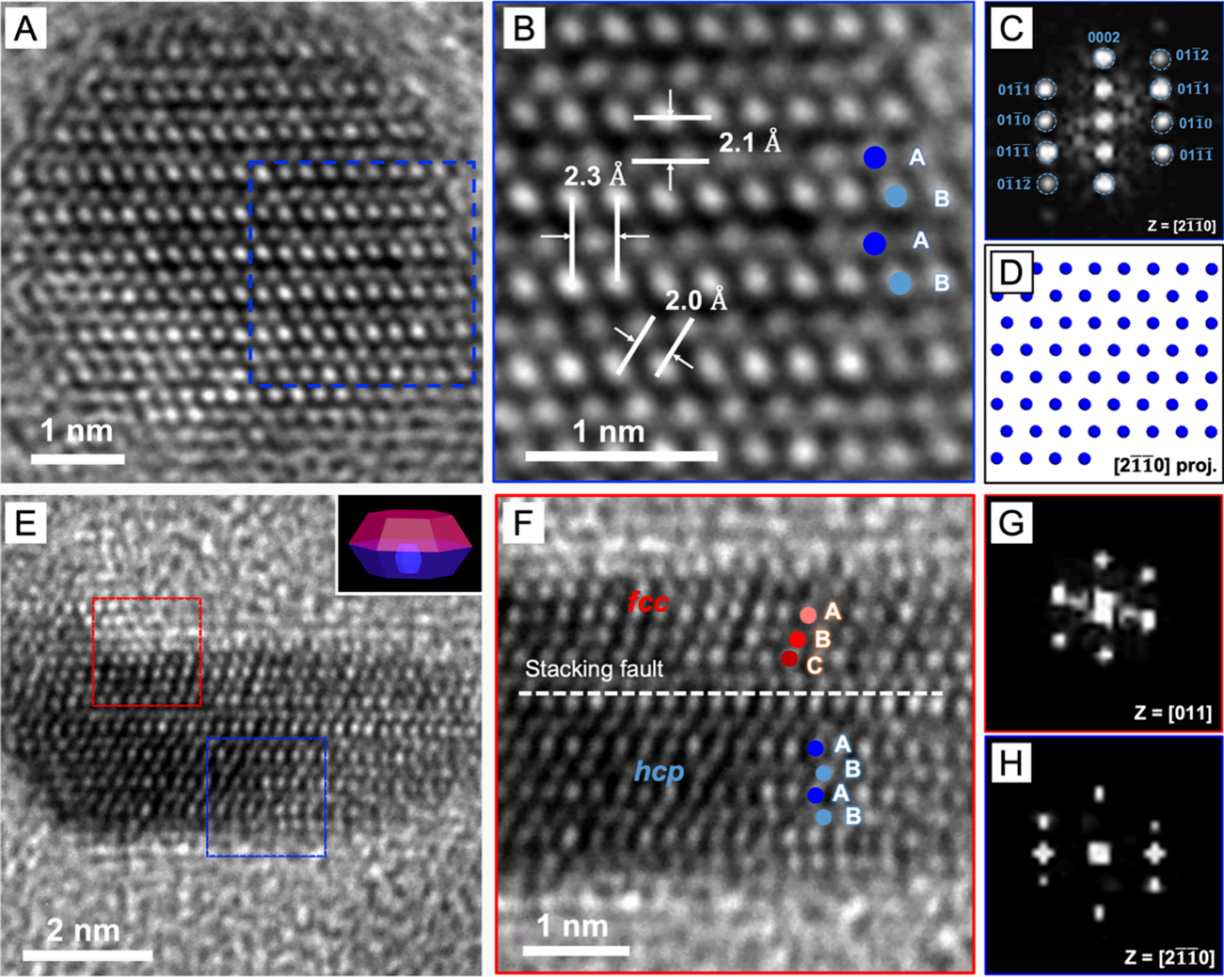
(A–D) Characterizations of Ru_*hcp*_@Ru_*hcp*_ nanocrystals: (A) HRTEM image, (B) atomic resolution HRTEM image, (C) the corresponding FFT pattern of the blue boxed region in A, and (D) a projected model of Ru atoms in the *hcp* structure viewed along the [21̅10] zone axis. (E–H) Characterizations of Ru_*hcp*_@Ru_*fcc*_ nanocrystals: (E) HRTEM image, (F) atomic resolution HRTEM image, and (G,H) the corresponding FFT patterns of the red and blue boxed regions in E, respectively. The inset in panel E shows a schematic illustration of the Ru_*hcp*_@Ru_*fcc*_ nanocrystal with a plate-like morphology.

We further confirmed the phase compositions of Ru_*hcp*_@Ru_*hcp*_ and Ru_*hcp*_@Ru_*fcc*_ nanocrystals by HRTEM. The HRTEM image of an individual Ru_*hcp*_@Ru_*hcp*_ nanocrystal and the corresponding magnified image of the surface region clearly show the distinctive “ABABAB” atomic stacking sequence of the *hcp* structure along the close-packing direction (Figure 3A, B). The lattice fringe spacings of 2.1, 2.3, and 2.0 Å correspond to the (0002), (101̅0), and (101̅1) planes of *hcp*-Ru. The FFT pattern obtained from the surface region, marked by a blue box, fits the *hcp* lattice viewed along the [21̅10] zone axis (Figure 3C). This result is also in agreement with the projected atomic model of *hcp*-Ru from the same perspective (Figure 3D). On the other hand, the HRTEM image and the corresponding magnified image of an individual Ru_*hcp*_@Ru_*fcc*_ nanocrystal revealed a plate-like morphology and an incomplete *fcc*-Ru shell, demonstrating the existence of an *hcp*/*fcc* heterophased interface (Figure 3E, F). The distinct atomic arrangements in the *hcp* phase (ABABAB) and *fcc* phase (ABCABC) could be clearly resolved along the [21̅10] and [011] zone axes, respectively, perpendicular to the basal plane of the nanoplate. The interface between the *hcp* and *fcc* phases, indicated by a stacking fault, could be formed due to the symmetry alignment (*C*_3_) of the *fcc*-{111} and *hcp*-{0001} facets.^18,20,27^ The FFT pattern taken from the middle area of the core (blue boxed) matches the diffraction pattern of the *hcp* phase along the [21̅10] zone axis, while the FFT pattern acquired from the shell region (red boxed) is consistent with the diffraction pattern of the *fcc* phase along the [011] zone axis (Figure 3G, H). The deposition of *fcc*-Ru overlayers predominantly occurred on the top half of the Ru_*hcp*_ seed, and this could be attributed to an asymmetrical growth mode initiated following the formation of the *fcc* phase in the first few layers. This asymmetrical deposition of the *fcc*-Ru shell likely resulted from a combination of factors, including the preferential alignment of crystal facets at the *hcp*/*fcc* interface, the high surface energy introduced by stacking faults, and the limited supply of the precursor. Taken together, the type of polyol still plays an essential role in determining the phase of the deposited Ru even in the case of seed-mediated growth.

To further validate the observed effects of polyol on the crystal phase taken by the Ru shell, we conducted a second round of overgrowth from the as-obtained Ru_*hcp*_@Ru_*hcp*_ and Ru_*hcp*_@Ru_*fcc*_ nanocrystals. This study aimed to discern whether the dominance of the polyol over the template was somewhat related to the small size (3.1 nm) of the initial seed. The second round of overgrowth was conducted by reducing the amount of Ru(acac)_3_ precursor from 5 to 2.5 mg while keeping all other parameters consistent with the protocol for the first round. Figure S4 shows typical TEM images of a series of nanocrystals obtained by depositing an additional Ru shell on Ru_*hcp*_@Ru_*hcp*_ or Ru_*hcp*_@Ru_*fcc*_ nanocrystals in the cases of EG and TEG, respectively. All products exhibited similar sizes of *ca*. 7 nm, which, compared to the seed size of *ca*. 5 nm, suggested the additional deposition of a Ru shell. The absence of particles smaller than the seeds or with significantly different morphologies implied the dominance of heterogeneous nucleation and layer-by-layer growth of the Ru atoms.

**Figure 4:**
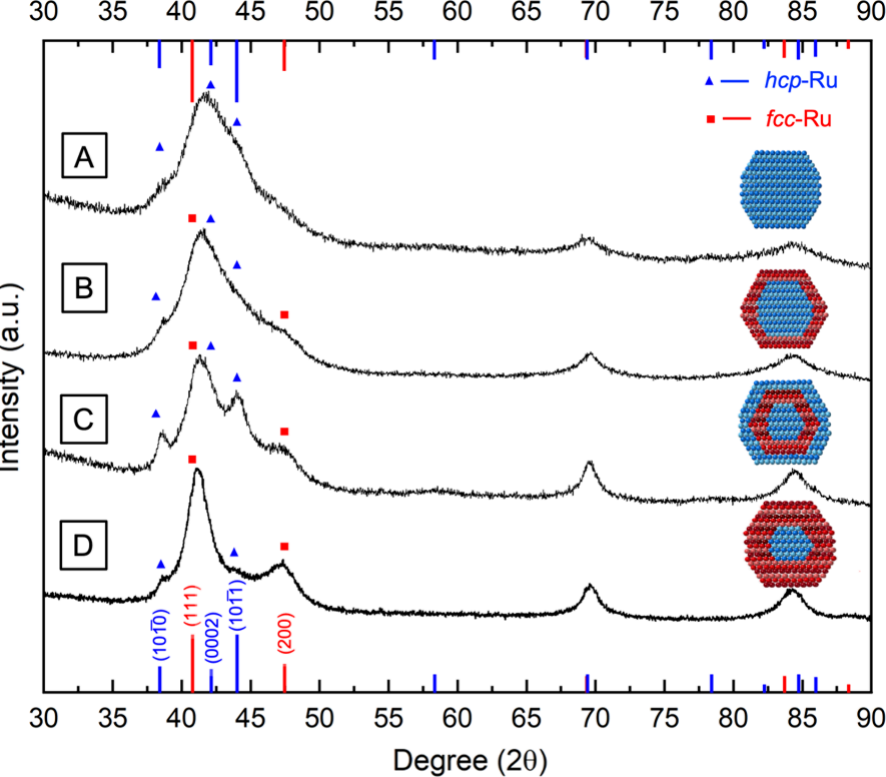
XRD patterns of the 7 nm Ru nanocrystals with an *hcp* or *fcc* phase in the outermost shell obtained from additional overgrowth on (A, B) Ru_*hcp*_@Ru_*hcp*_ and (C, D) Ru_*hcp*_@Ru_*fcc*_ seeds in different types of polyols: (A, C) EG and (B, D) TEG. The red and blue lines correspond to the characteristic peaks of *fcc*-Ru (JCPDS No. 01–088–2333) and *hcp*-Ru (JCPDS No. 06–0663), respectively. The insets in panels A and D are the corresponding atomic models.

The homo- or heterophased structure of the 7 nm Ru nanocrystals was validated by analyzing their XRD patterns. For the sample synthesized in EG from the Ru_*hcp*_@Ru_*hcp*_ template (referred to as Ru_*hcp*_@Ru_*hcp*_@Ru_*hcp*_), the XRD pattern displayed three more resolved peaks at 2θ of 38.4°, 42.1°, and 44.0°, corresponding to (101̅0), (0002), and (101̅1) planes of *hcp*-Ru, respectively. Although the peaks were not sharply defined, their higher degree of separation is indicative of a high crystallinity and a consistent homophase in the entire particle (Figure 4A). Conversely, the XRD pattern of the products from the Ru_*hcp*_@Ru_*hcp*_ seed and TEG (denoted Ru_*hcp*_@Ru_*hcp*_@Ru_*fcc*_) presents an intriguing mix of the *hcp* and *fcc* phases. Besides the stronger intensity of the characteristic peaks of *hcp*-Ru (*i.e*., the (101̅0) peak at 38.6°) due to the size increase, a weak peak was observed around 47.4°, which can be assigned to the (200) diffraction of *fcc*-Ru (Figure 4B). Additionally, the main peak slightly shifted to 2θ of 41.4°, situated between the reference peaks of *fcc*-Ru(111) and *hcp*-Ru(0002). These results suggested that the interior maintained its initial *hcp* structure while the outermost shell adopted the *fcc* structure as directed by TEG.

The sample prepared from the Ru_*hcp*_@Ru_*fcc*_ seed in EG (denoted Ru_*hcp*_@Ru_*fcc*_@Ru_*hcp*_) offered yet another layer of complexity in the XRD analysis. The three characteristic peaks in the region of 38–45° reveal the dominance of the *hcp* phase, attributed to the outermost shell and the core (Figure 4C). However, the shoulder *fcc*-Ru(200) peak at 2θ of 47.4°, coupled with the shift of the main peak to the region between *fcc*-Ru(111) at 40.8° and *hcp*-Ru(0002) at 42.1°, confirmed the existence of the *fcc* phase in the intermediate shell, albeit at a low intensity due to its minor contribution to the entire particle. As for the sample prepared from the Ru_*hcp*_@Ru_*fcc*_ seeds in TEG (denoted Ru_*hcp*_@Ru_*fcc*_@Ru_*fcc*_), the XRD pattern displayed dominant *fcc*-Ru characteristics with sharper (111) and (200) peaks due to the increased crystallite size and larger contribution from the *fcc* phase (Figure 4D). The significant shift of the primary peaks toward the characteristic peak positions of *fcc*-Ru(111) and *fcc*-Ru(200) diffractions is indicative of the transformative effect of TEG to favor the formation of *fcc*-Ru. The residual *hcp* peaks from the core could still be observed but notably diminished, further underlining the overpowering influence of the polyol on the crystal phase, even in the presence of a Ru_*hcp*_ seed as the template.

**Figure 5:**
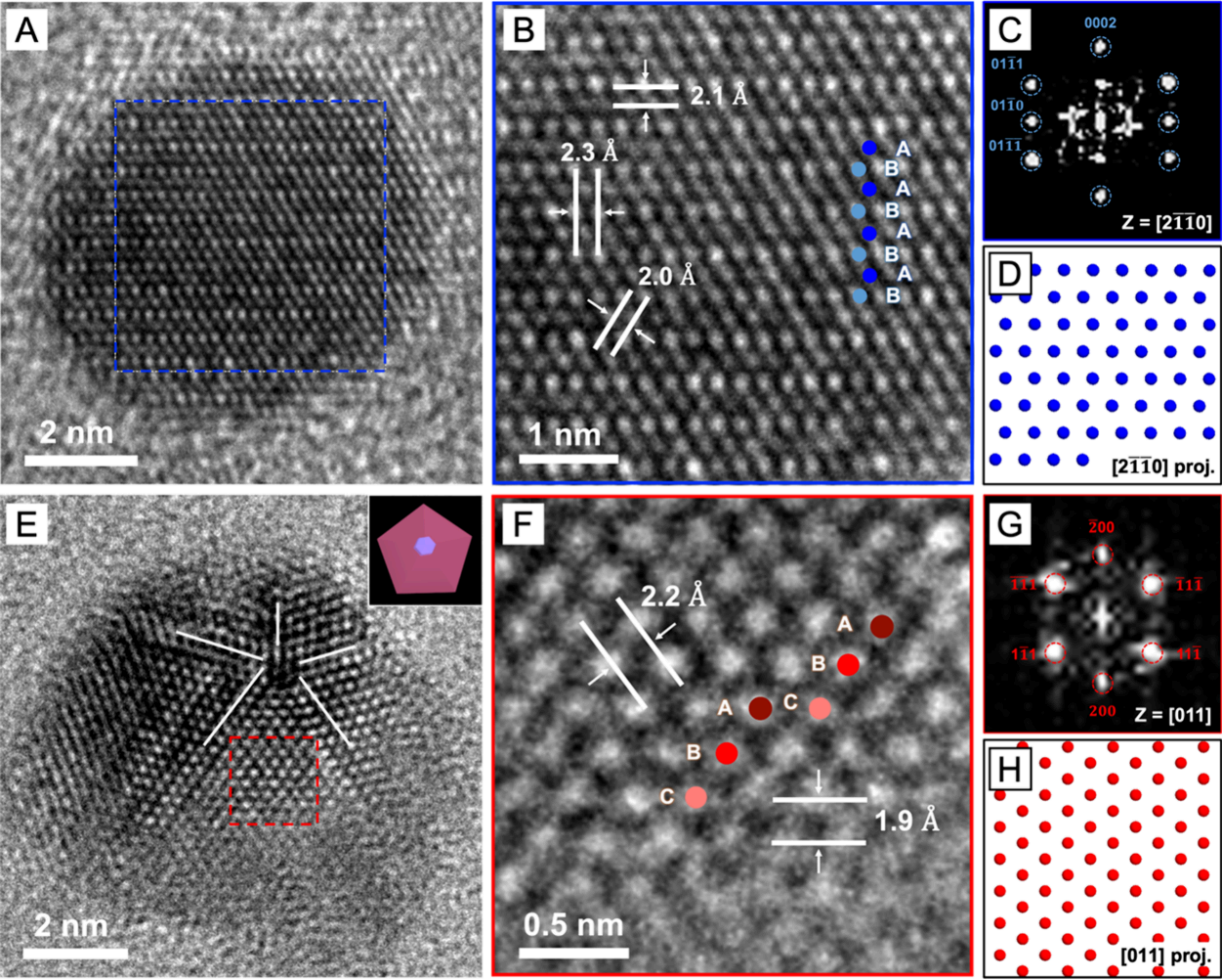
(A–D) Characterizations of Ru_*hcp*_@Ru_*hcp*_@Ru_*hcp*_ nanocrystals: (A) HRTEM image, (B) atomic resolution HRTEM image, (C) the corresponding FFT pattern of the blue boxed region in A, and (D) a simulated model for the atomic arrangement of *hcp* structure viewed along the [21̅10] direction. (E–H) Characterizations of Ru_*hcp*_@Ru_*fcc*_@Ru_*fcc*_ nanocrystals: (E) HRTEM image, (F) atomic resolution HRTEM image, (G–H) the corresponding FFT patterns of the red boxed region in E, and (D) a projected model of Ru atoms in the *fcc* structure viewed along the [011] direction. In panel E, the white lines indicate twin boundaries while the inset shows a schematic of the Ru_*hcp*_@Ru_*fcc*_@Ru_*fcc*_ nanocrystal with a 5-fold twin structure.

To support the XRD data, we employed HRTEM to confirm the crystal phases of the products obtained from the second round of overgrowth. Given the emphasis on understanding the influence of the polyol and the need for clarity, we prioritized the analysis of two representative samples: Ru_*hcp*_@Ru_*hcp*_@Ru_*hcp*_ exemplifying a homophased structure and Ru_*hcp*_@Ru_*fcc*_@Ru_*fcc*_ with the metastable *fcc* phase in dominance to highlight the polyol-induced phase transitions. Figure 5A, B shows the HRTEM image and the corresponding atomic resolution image of Ru_*hcp*_@Ru_*hcp*_@Ru_*hcp*_ nanocrystals. The lattice fringe spacings align well with the expected interplanar spacings for the *hcp*-Ru plane. A magnified view of the surface region reveals continuous ABABAB atomic packing indicative of the *hcp* phase throughout the particle. This observation is in accord with our XRD findings, reaffirming the homophased structure. The FFT analysis of this region yields a diffraction pattern that corresponds to the *hcp* crystal structure viewed along the [21̅10] zone axis (Figure 5C, D). In contrast, the HRTEM image of a Ru_*hcp*_@Ru_*fcc*_@Ru_*fcc*_ nanocrystal shows a 5-fold twinned structure typically observed in *fcc* nanocrystals (Figure 5E). The *hcp*-Ru core could not be clearly resolved in the HRTEM image due to its small size and the dominance of the *fcc*-Ru shell in the second round of overgrowth. Besides the ABCABC atomic packing, the lattice fringes aligning with *fcc-*Ru interplanar spacings also dominate each tetrahedral subunit of the multiply twinned nanocrystal (Figure 5F). The FFT pattern of one tetrahedral subunit also exhibits diffraction spots corresponding to the *fcc*-Ru phase viewed along the [011] direction (Figure 5G, H). Collectively, these findings attest to the dominant role played by the polyol in dictating the crystal phase of the additionally deposited Ru. Choosing EG or TEG consistently resulted in the formation of a Ru shell with the native *hcp* and metastable *fcc* phases, respectively. This outcome was observed regardless of the size and initial phase of the Ru nanocrystal seeds.

Despite the profound influence of the polyol, it is worth noting that the seed still plays a pivotal role in the synthesis. As a noble metal high in cohesive energy, Ru nanoparticles tended to show very small sizes (typically, < 5 nm) in previous studies that employed a one-pot, solution-phase synthesis.^24,27,52^ The presence of seeds herein served as catalysts to drive heterogeneous nucleation and further growth for the formation of Ru nanocrystals with larger sizes. The appropriate size of the seeds would also ensure the balance between the bulk and surface energies to favor the metastable *fcc*-Ru phase when TEG was used as the polyol. Overall, our experimental results highlight the intricate interplay among the polyol, seed, and deposition conditions in determining the crystal phase taken by the final products. Polyol selection has emerged as a key factor in the phase-controlled synthesis of Ru nanocrystals. Several plausible mechanisms might underpin this phase dependence on the chemical environment, such as the preferential surface capping of EG or TEG toward *hcp* or *fcc* facets, the decrease in reduction kinetics of metal precursor due to reduced electron transfer efficiency and thus weaker reducing power of long-chain polyols, or other types of molecular interactions.^16,27,35,53^ While these hypotheses provide avenues for exploration, a more comprehensive understanding at the atomic and/or molecular level is necessary to achieve rational synthesis of Ru nanocrystals with the desired phases in high purity.

### Quantitative Analysis of the Reduction Kinetics and Pathways Involved

The formation of a nanocrystal with a specific phase is intrinsically linked to its nucleation and growth details, which are in turn determined by the reduction and deposition kinetics of the metal precursor.^8,49,53^ To gain quantitative insights into the transition from *hcp*-Ru core to *fcc*-Ru shell when substituting EG with TEG as the polyol, we conducted a kinetic measurement of the reduction rate constants of Ru(acac)_3_ precursor at 180 °C, under different reaction conditions, and in the presence of the 3.1 nm Ru_*hcp*_ seeds. Figure S5A shows plots of the concentrations of the remaining precursor as a function of reaction time for one-shot syntheses involving EG and TEG, respectively, which were measured using inductively-coupled plasma mass spectrometry (ICP-MS). Even when initiated at the same concentration, the precursor was quickly depleted within the first 10 min in the EG-based synthesis, whereas nearly 50% of the precursor still remained after 50 min in the TEG system, suggesting a large difference in reduction kinetics.

By approximating the reaction kinetics as a pseudo-first-order reaction and calculating the slopes of the linear regression lines, the reduction rate constants (*k*) were determined as 0.18 s^–1^ and 0.015 s^–1^, respectively (Figure S5B). Combining with the initial concentration of Ru(acac)_3_ (1.14 mM), we deduced the initial reaction rates as 2.1 × 10^–4^ M s^–1^ for EG and 1.7 × 10^–5^ M s^–1^ for TEG, corresponding to the formation of *hcp*- and *fcc*-Ru overlayers, respectively. This substantial difference in initial reduction rate, spanning over 1 order of magnitude, further corroborates the more rapid reduction process in EG compared to TEG. On the basis of *k* values derived from one-shot injections, we further simulated the instantaneous concentrations and thus reduction rates of Ru(acac)_3_ precursor under dropwise syntheses as described in the standard protocol (Figure S5C). In the EG-based system, the reduction rate of the precursor reached a maximum level of 10.6 × 10^–6^ M min^–1^ during the first 15 min, which then transitioned to a steady state as the reaction proceeded. In contrast, the reduction in TEG proceeded at a markedly slower rate without the sharp initial peak or high-frequency oscillations seen in EG, implying a more gradual increase in the reduction rate. From the kinetic measurements for one-shot and dropwise injections, the precursor consistently exhibited a faster reduction in EG compared to TEG.

Our prior studies have established that the reduction pathway undertaken by a salt precursor is contingent upon the reduction kinetics involved.^47^ Fast kinetics typically leads to solution reduction, while slow kinetics favor reduction on the surface of existing seeds through an autocatalytic process. Based on the different kinetic profiles observed in the two systems, we postulated that the conversion from precursor to atoms predominantly occurs via two distinct pathways: solution reduction in EG versus surface reduction in TEG (Figure S5D). In the EG-based synthesis, the solution reduction manifests as an initial spike in reduction rate, followed by a gradual plateau after 15 min, indicative of a fast conversion to Ru atoms in the solution that transitions to a steady state, where the rate of atomic formation is balanced by the rate of atomic addition onto the surface of existing seeds through heterogeneous nucleation. However, the slower initial reduction rate observed in TEG suggests a surface reduction pathway, where the precursor is first adsorbed onto the seed and then reduced to atoms for the continuous growth of the particle. The gradual increase in the reduction rate over the course of TEG-based synthesis before reaching a steady state could be ascribed to the continuous growth of the seed and thus availability of a larger surface serving as a catalyst for the reduction process. Combining the synthetic and kinetic results, the fast reduction kinetics in EG corresponds to solution reduction, leading to the formation of the *hcp*-Ru shell, while a slow reduction kinetics in TEG favors surface reduction and the formation of the *hcp*-Ru shell.

### Mechanistic Investigation by First-Principles DFT Calculations

We used DFT-based calculations to elucidate the atomic-scale interactions that govern the phase-selective growth observed experimentally. Previous studies indicate that RuO_2_ is formed as an intermediate during the early stages of Ru(acac)_3_ decomposition in the presence of oxygen or oxygen-containing species,^54−57^ serving as a transition point between the Ru(III) precursor and Ru atoms. As such, it is not unreasonable to focus our analyses on interactions involving RuO_2_ rather than Ru(acac)_3_. In our DFT model, we assume that the polyol is not directly involved in the decomposition of Ru(acac)_3_, but that it participates in the subsequent reduction of RuO_2_. Significantly, the reduction kinetics of the precursor play a vital role in dictating the formation of different phases of Ru overlayers on the Ru template.

**Figure 6:**
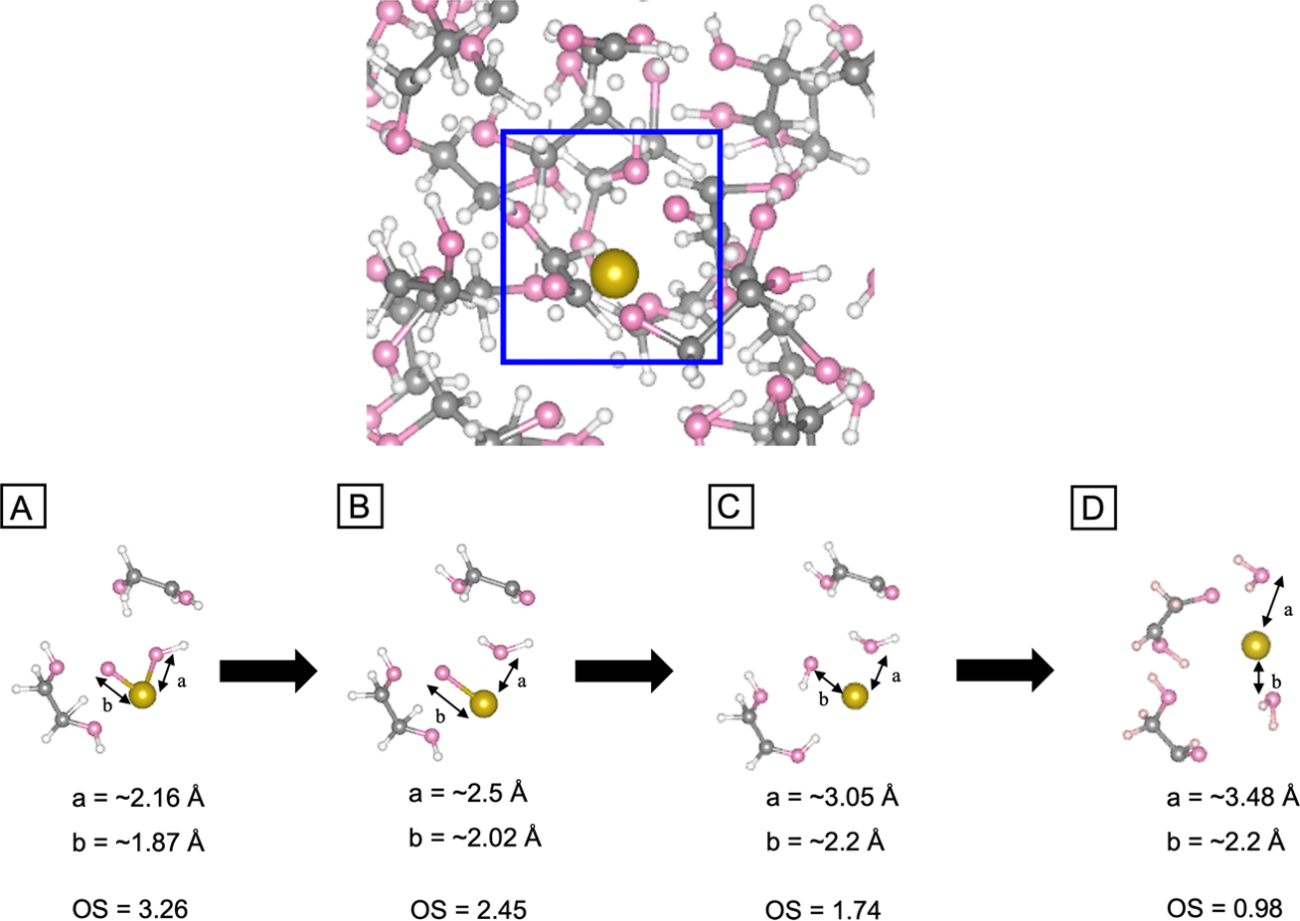
Snapshots from an AIMD simulation of the reduction of RuO_2_ by EG. The blue box depicts the region where Ru reduction occurs and (A–D) show the progress of RuO_2_ reduction, together with the OS of Ru. Yellow: Ru in EG; pink: oxygen; gray: carbon; white: hydrogen.

We first investigated the solution-phase reduction of a single RuO_2_ by EG and TEG. We studied a variety of solution temperatures (see the Experimental Section) and found that reduction of RuO_2_ by EG occurred over the *Ab Initio* Molecular Dynamics (AIMD) time scale at 2000 K—the highest temperature examined for which pyrolysis did not occur. Figure 6 outlines the observed reduction mechanism. In the first step (Figure 6A), EG donated a hydrogen atom (from a carbon) to RuO_2_, creating a hydroxyl group associated with the Ru atom (O–Ru–OH) and ethane-1,2-diol. The Ru–O bond length in the hydroxyl group increased from 1.68 Å in RuO_2_ to 2.16 Å and the increased bond length could be interpreted as RuO_2_ becoming partially reduced by EG. In Figure 6B, ethane-1,2-diol was reduced to glycolaldehyde with the loss of another hydrogen atom from the OH group. The hydrogen atom created a water molecule by making a bond with the hydroxyl group associated with the Ru atom. The newly created water left the Ru atom with a Ru–O distance of *ca*. 2.5 Å. In Figure 6C, another EG molecule donated a hydrogen atom, creating a hydroxyl group around the Ru atom in RuO and ethane-1,2-diol. In Figure 6D, the new ethane-1,2-diol surrounding Ru was reduced to glycolaldehyde with the loss of another hydrogen atom from the OH group, creating another water molecule. On the other hand, RuO_2_ remained intact during our AIMD simulation in TEG at the same temperature over the same time scale (Figure S7).

To quantify the reduction of RuO_2_ by EG, we created a correlation between the oxidation state (OS) and the Bader charge of Ru using known Ru compounds. In Figure S8, we plot this correlation. After our AIMD simulation at 2000 K, the RuO_2_ molecule stayed intact in TEG solution, showing OS = 4.06 and OS = 4.18 for Figure S7A and B, respectively, which is similar to an RuO_2_ molecule in the gas phase (OS = 4.43). On the other hand, the final Ru species was partially reduced by EG. In Figure 6A, the OS was partially reduced to 3.26 as a hydrogen atom in EG adsorbed on RuO_2_, creating a hydroxyl group associated with the Ru atom (O–Ru–OH) and ethane-1,2-diol. In Figure 6B, the OS was further reduced to 2.45 by donating another hydrogen from EG and creating another water surrounding Ru. In Figure 6C, the OS decreased to 1.74 by another EG donating hydrogen, creating another hydroxyl group associated with the Ru atom. Finally in Figure 6D, the OS decreased to 0.98 by creating another water molecule while making a bond with the hydroxyl group associated with the Ru atom. The OS seemed to be affected by the resulting molecules surrounding the Ru atom in solution. For example, the OS decreased from 2.45 to 1.74 when the distance between Ru atom and resultant molecules in solution was increased (Figure 6B, C). However, the OS did not fully decrease to zero when both oxygen atoms left Ru (Figure 6D). This may be due to limitations of the semilocal PBE functional employed in our DFT calculations^58^ or charge self-regulation.^59^ Nevertheless, it is evident in the AIMD simulations that EG can remove oxygen atoms from RuO_2_, resulting in solution-phase reduction.

When RuO_2_ is completely reduced by EG, the resulting Ru atoms will prefer *hcp* binding sites on *hcp*-Ru(0001) in the presence of EG. As a check of the hypothesis, we ran various DFT calculations to probe the influence of EG on Ru binding to Ru(0001), and it was found that the binding preference of the Ru atom in EG was the same as that in vacuum (see Figures S9).

**Figure 7:**
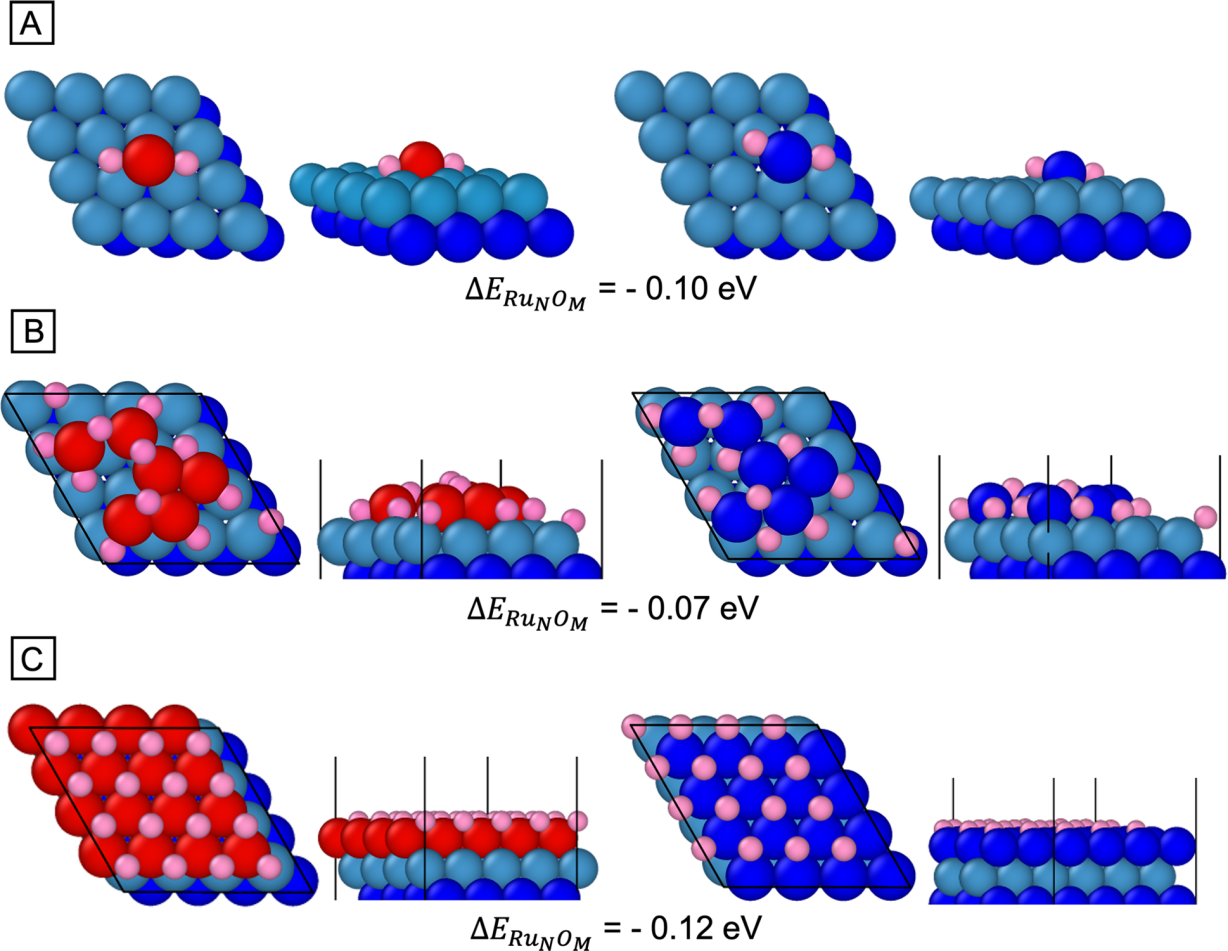
Top and side view of Ru_*N*_O_*M*_ at *fcc* and *hcp* site on *hcp*-Ru(0001). Ru*_N_*O_*M*_, given by Equation S1, is indicated. (A) one RuO_2_ deposited with Ru at *fcc* and *hcp* sites; (B) RuO_2_ with a coverage (θ) of 3/8 at *fcc* and *hcp* sites; (C) Ru_*N*_O_*M*_ where Ru atoms are at *fcc* (left) and *hcp* (right) and oxygen atoms are at *hcp* (left) and *fcc* (right) sites on *hcp*-Ru (0001). Sky and blue: *hcp*-Ru; red: *fcc*-Ru; pink: oxygen.

Since TEG did not reduce RuO_2_ in solution (Figure S7), we argued that RuO_2_ would be deposited on the surface of an *hcp*-Ru seed in the case of TEG. Using evidence from a previous DFT study,^60^ we proposed that the O in RuO_2_ would affect the binding site of Ru. We investigated the preferred site and binding configuration of RuO_2_ using different structures in DFT. First, we calculated Δ*E*_Ru_*N*_O_*M*__ using Equation S1, see the Supporting Information, for four configurations of a single RuO_2_ on *hcp*-Ru(0001), with the Ru atom residing on the *fcc* and *hcp* sites, respectively. These configurations were optimized to four configurations, as shown in Figure S10. For all configurations, the results indicated that a single RuO_2_ preferred to bind to the *fcc* site on *hcp*-Ru (0001) (negative Δ*E*_Ru_*N*_O_*M*__ from Equation S2). Figure 7A shows the preferred binding site and configuration for RuO_2_ on the *fcc* and *hcp* sites of *hcp*-Ru(0001).

We subsequently probed four different RuO_2_ coverages (θ, where θ = the number of Ru atoms divided by the number of Ru atoms in the top surface layer), with RuO_2_ initially on *fcc* and *hcp* binding sites on Ru(0001). In these calculations, we observed that RuO_2_ became progressively more disordered on the Ru(0001) template as the coverage increased (Figure S11), with O atoms leaving the original RuO_2_ to assume various binding configurations on the surface. We were able to obtain a maximum coverage of RuO_2_ at θ = 3/8, as steric constraints occurred at higher coverages. For all of these studied coverages, the *fcc* sites were energetically preferred over *hcp* sites for the Ru atoms (Figure S11). Figure 7B shows a snapshot for θ = 3/8. In calculations with these disordered layers, we found that TEG was able to *spontaneously* remove the adsorbed oxygen during DFT optimization, as we discuss below. Thus, we considered that additional RuO_2_ could adsorb to the point at which we achieved a single *fcc* layer of Ru on *hcp*-Ru(0001) with a complete layer of adsorbed O, as shown in Figure 7C. Our results also indicate that the *fcc*-Ru layer is preferred over the *hcp*-Ru layer for this configuration.

**Figure 8:**
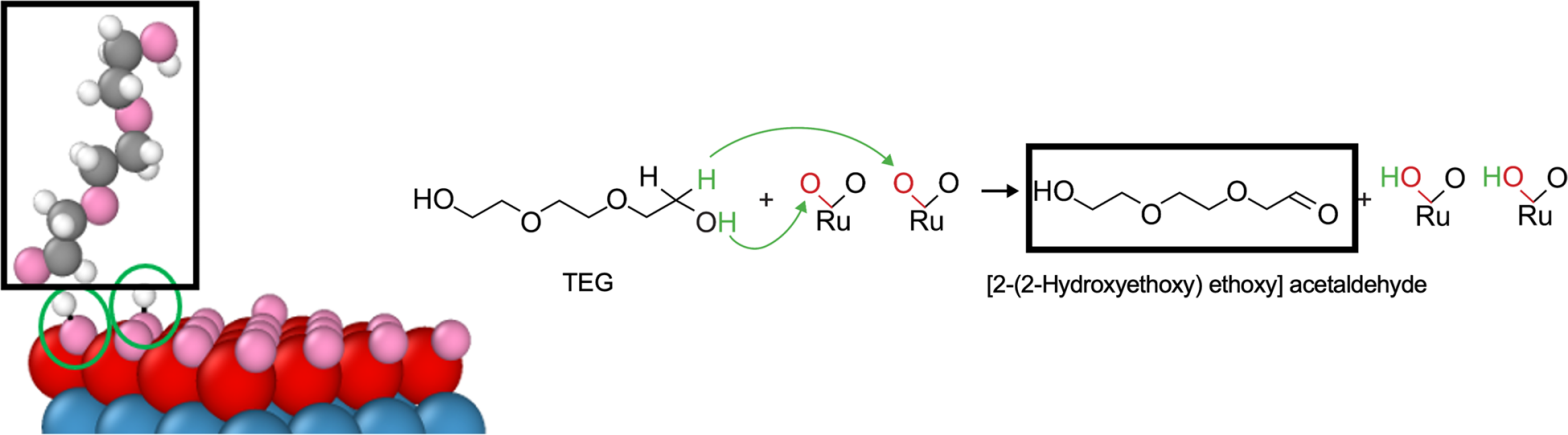
Snapshot of final configurations and the detailed mechanism involving the removal of hydrogen atoms from TEG. The green circles indicate the created hydroxyl groups by removal of hydrogen atoms from TEG. Sky and blue: *hcp*-Ru seed; red: *fcc*-Ru; pink: oxygen; gray: carbon; white: hydrogen.

Though our calculations indicate that TEG can reduce any of the RuO_2_ layers shown in Figure S11, it is advantageous to investigate the reduction of the high-symmetry layer in Figure 7C, where there is a one-to-one ratio of Ru to O. First, TEG chemisorbed onto the layer in Figure 8 with its long axis perpendicular to the surface—when TEG was placed in a configuration with its long axis parallel to the surface, it was physically adsorbed. We considered four different locations for the terminal OH group in TEG with respect to the surface, and these are shown in Figure S12A. After geometry optimization, we found TEG could remove the oxygen atoms from Ru_*N*_O_*M*_ through the hydrogens *spontaneously* leaving the TEG molecule (Figure 8 and Figure S12B).

For each of the four different locations of TEG adsorption in Figure S12A, hydrogen atoms on both the terminal OH and a neighboring terminal C in TEG left *spontaneously* during structural optimization in DFT at zero K and created two adsorbed hydroxyl groups with O atoms on the surface by oxidizing TEG and creating [2-(2-hydroxyethoxy) ethoxy] acetaldehyde. The resulting hydroxyl groups could also be *spontaneously* removed by an 8-hydroxyl-3,6-dioxaoctanal molecule, an oxidized TEG molecule that was observed in our AIMD simulation for TEG (Figure S13). We argue that this sequence of hydroxylation and dehydroxylation would continue until all the oxygen atoms were removed from the Ru surface. Unlike TEG, EG was not chemisorbed to the surface during DFT optimization. In Figure S14, we decomposed *E*_bind_ (binding energy of EG to the Ru_*N*_O_*M*_ surface) into two contributions: a short-range contribution (*E*_electronic_) and a long-range vdW interaction (*E*_vdW_). Based on our binding energy calculations, the contribution from *E*_vdW_ was 80–90% of the total *E*_bind_, indicating that EG was weakly bound to the surface. We also observed that EG did not lose its hydrogen spontaneously during geometry optimization, unlike TEG. Therefore, our calculations suggested that EG did not readily assist in the removal of oxygen atoms from the Ru surface.

After the oxygen atoms had been removed by TEG, *fcc*-Ru remained metastable during the DFT geometry optimization. We investigated the growth of a second layer of Ru, assuming all of the oxygen atoms in the first *fcc*-RuO_2_ overlayer had been removed during surface reduction by TEG. First, we deposited one RuO_2_ on the first *fcc-*Ru overlayer (Figure S15). We started with six different initial configurations for RuO_2_. After geometry optimization, we compared the binding energies of RuO_2_ on the *fcc*-Ru overlayer. Figure S15 shows the lowest energy configuration, with RuO_2_ residing at an *fcc* or *hcp* site. Our results indicated that *fcc-*RuO_2_ was preferred over *hcp-*RuO_2_ by *ca*. 0.03 eV.

We also deposited different coverages (θ) of Ru_*N*_O*_M_* as a second Ru overlayer (Figure S16). As for the first Ru layer, we obtained a maximum coverage of RuO_2_ at θ = 3/8. Based on relative binding energy calculations using Equation S1, *fcc-*Ru_*N*_O_*M*_ was preferred over *hcp-*Ru_*N*_O_*M*_. At the maximum coverage (θ) of RuO_2_, a second *fcc*-RuO_2_ overlayer was preferred over the *hcp*-RuO_2_ overlayer by *ca*. 0.38 eV (Figure S16), compared to a first *fcc*-RuO_2_ overlayer preference over *hcp*-RuO_2_ only by *ca*. 0.07 eV (Figure S11). Next, we deposited more Ru_*N*_O_*M*_, with oxygen adsorption occurring exclusively at *hcp* or *fcc* sites (Figure S17). The *fcc-*RuO_2_ was still preferred over *hcp* sites by *ca*. 0.02 eV. When we deposited Ru instead of RuO_2_, Ru preferred *hcp* sites over *fcc* by *ca* = 0.19 eV (Figure S18). Altogether, these results suggested that RuO_2_ overlayers could be subsequently deposited on the *fcc*-Ru seed, following surface reduction by TEG. This is consistent with the experimental finding that a second overlayer of Ru would also take the *fcc* phase when growth occurs in TEG.

**Figure 9:**
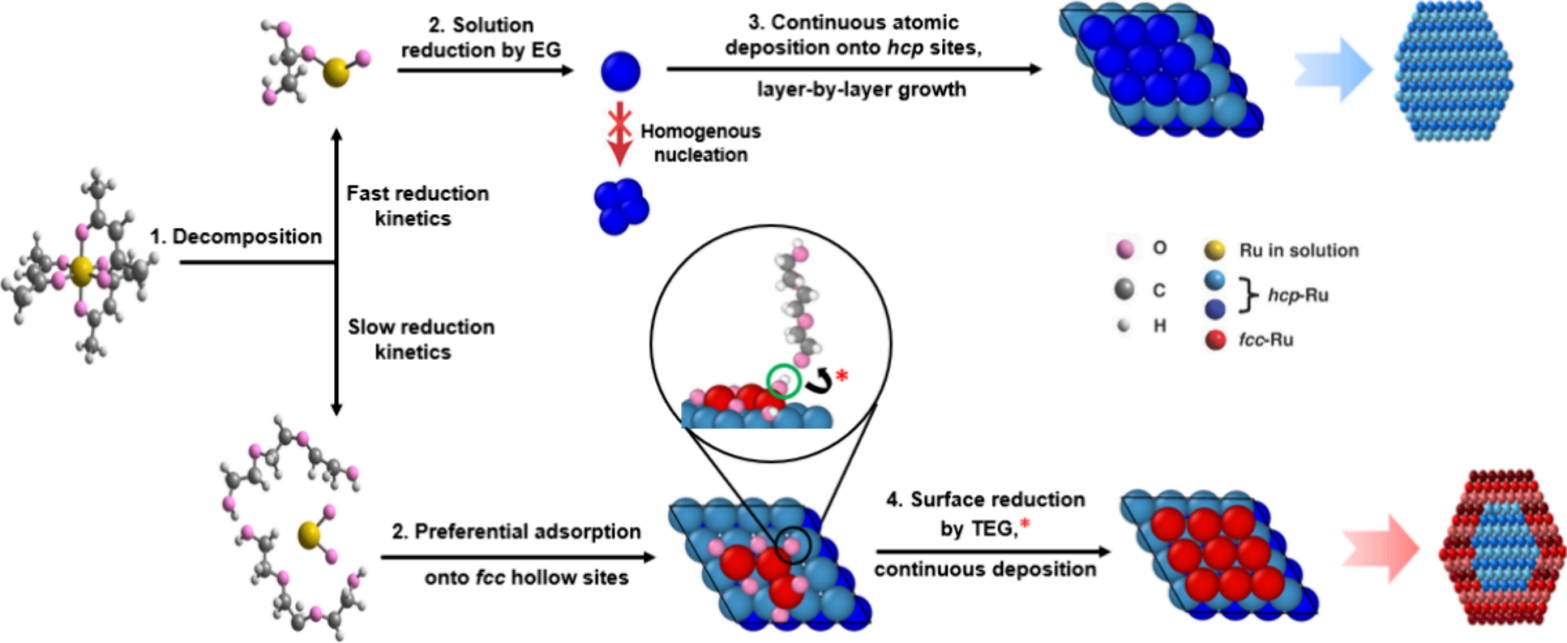
Mechanism of the effect of reduction kinetics on the phase-selective epitaxial growth of Ru overlayers based on both computational and experimental results.

Collectively, our calculations support the experimental findings that the different reduction kinetics of the precursor with the chosen polyol play a dominant role in dictating the Ru crystal structure via distinct reduction pathways (Figure 9). Specifically, TEG reduces the precursor by removing the oxygen atoms in *fcc*-RuO_2_ at a slow reduction rate, leading to the *fcc*-Ru crystal phase. In contrast, EG has the capability to reduce the precursor in the solution at a fast reduction rate, favoring the formation of the *hcp*-Ru phase.

## Conclusion

In summary, we have systematically investigated the use of seed-mediated growth to fabricate Ru nanocrystals with tunable sizes of 3–7 nm and distinct patterns (either homo- or heterophased) of crystal phases by manipulating the polyol serving as a solvent and a reducing agent in each round of growth. By keeping all experimental conditions except the type of polyol fixed, we found that EG exclusively yielded the thermodynamic *hcp* phase, whereas TEG favored the formation of the metastable *fcc* phase, regardless of the size and crystal structure of the seeds involved. While both the chemical environment and template could affect the outcome of a phase-selective epitaxial growth, the chemical environment tended to play a more important role in controlling the reduction kinetics of the precursor and thereby dictating the polymorphism (*hcp versus fcc*) of the Ru overlayers.

Our theoretical analyses, based on both DFT and AIMD, supported the experimental findings by attributing the polyol-based phase control to its different interactions with both the seed surface and the precursor, which directly influenced the reduction pathway. Specifically, the reduction could follow two different pathways, resulting in distinct packing patterns for the Ru atoms. Under fast reduction kinetics due to the strong reducing power of EG, solution reduction dominated, where different Ru-based complexes were formed as intermediates and then reduced to Ru atoms in solution, which were preferentially deposited on the *hcp* sites of the *hcp*-Ru seeds. In contrast, a slower reduction kinetics due to a weaker reducing power of TEG favored the surface reduction pathway, initiating with the formation of a RuO_2_ layer that preferentially adsorbed on the *fcc* sites, followed by the removal of oxygen by TEG to generate *fcc*-Ru overlayers. The insights into the mechanisms of reduction kinetics in different polyols not only offer guidelines for achieving Ru nanocrystals with a desired phase in high purity but also shed light on the rational synthesis of phase-controlled metal nanocrystals. Future studies are encouraged to extend this work to other metals, validating the universality and potential of reduction kinetics as a pivotal knob to maneuver the crystal phases of various types of nanocrystals.

## Supporting Information Available

Descriptions of synthetic protocols, computational details, additional TEM and HRTEM images, and XRD patterns of the initial Ru seeds and samples prepared under different conditions, as well as results from additional computational analyses (PDF)
