# Supplementary material for: Elucidating the Role of Reduction Kinetics in the Phase-Controlled Growth on Preformed Nanocrystal Seeds: A Case Study of Ru
Source: J Am Chem Soc. 2024 Mar 30;146(17):12040–52. doi: 10.1021/jacs.4c01725 (PMC11066843; doi:10.1021/jacs.4c01725)
Supplement: Supplementary file 1 — ja4c01725_si_001.pdf [file ja4c01725_si_001.pdf]

Supporting Information

Elucidating the Role of Reduction Kinetics in the Phase-Controlled Growth on Preformed Nanocrystal Seeds: A Case Study of Ru

Quynh N. Nguyen,[†] Eun Mi Kim,[‡] Yong Ding,[§] Annemieke Janssen,[†] Chenxiao Wang,[†] Kei Kwan Li,[†] Junseok Kim,[‡] Kristen A. Fichthorn,[‡,*] and Younan Xia[†,¶,*]

[†]School of Chemistry and Biochemistry, Georgia Institute of Technology, Atlanta, Georgia 30332, United States

[‡]Department of Chemical Engineering, The Pennsylvania State University, University Park, Pennsylvania 16803, United States

[§]School of Materials Science and Engineering, Georgia Institute of Technology, Atlanta, Georgia 30332, United States

[¶]The Wallace H. Coulter Department of Biomedical Engineering, Georgia Institute of Technology and Emory University, Atlanta, Georgia 30332, United States

[*]Address correspondence to fichthorn@psu.edu (for the computational study) and younan.xia@bme.gatech.edu (for the experimental study)

EXPERIMENTAL SECTION

Chemicals and Materials. Ruthenium(III) acetylacetonate ($Ru(acac)_3$, 97%), triethylene glycol (TEG, 99%), and poly(vinylpyrrolidone) (PVP, with an average molecular weight of 55k) were purchased from Sigma-Aldrich. Ethylene glycol (EG, 99%) was obtained from J. T. Baker. Ethanol (99.5%) and acetone (99.5%) were purchased from VWR. All chemicals were used as received. Aqueous solutions were prepared using deionized water with a resistivity of 18.2 MΩ·cm at room temperature.

Synthesis of Ru$_{hcp}$ Nanocrystal Seeds. The 3.1-nm Ru$_{hcp}$ nanocrystals serving as seeds in the first round of overgrowth were synthesized by slightly modifying a reported protocol.[1] In a typical synthesis, 7.5 mg of $Ru(acac)_3$ and 50 mg of PVP were mixed in 5 mL of EG hosted in a 20 mL glass vial. The vial was then heated in an oil bath held at 180 °C under magnetic stirring at 400 rpm. After 2 h, the reaction was quenched by immersing the container in an ice-water bath. For the overgrowth involving EG, the as-obtained suspension of Ru$_{hcp}$ nanocrystals in EG (*ca.* 1.35 mg/mL) was directly used without further treatment. When TEG was involved in overgrowth, the nanocrystals were crushed out with acetone (acetone/EG = 3:1, v/v), followed by centrifugation and washing three times with a mixture of acetone and ethanol (acetone/ethanol = 3:1, v/v). The solids were finally redispersed in TEG (*ca.* 1.35 mg/mL) for further use.

Synthesis of Ru$_{hcp}$@Ru$_{hcp}$ and Ru$_{hcp}$@Ru$_{fcc}$ Nanocrystals. In a typical synthesis, 1 mL of the as-synthesized Ru$_{hcp}$ nanocrystal seeds (dispersed in EG or TEG at *ca.* 1.35 mg/mL for the Ru$_{hcp}$@Ru$_{hcp}$ and Ru$_{hcp}$@Ru$_{fcc}$ samples, respectively) and 50 mg of PVP were transferred into a 20-mL glass vial. The mixture was heated at 180 °C for 10 min under magnetic stirring (400 rpm). Meanwhile, 5 mg of $Ru(acac)_3$ and 25 mg of PVP were dissolved in 10 mL of EG or TEG by heating at 90 °C for 15 min under magnetic stirring (400 rpm). The as-obtained reddish solution was added into the growth solution at a rate of 0.5 mL h^{-1} using a syringe pump. After all the precursor solution had been added, the reaction was allowed to proceed for another 2 h and then quenched with an ice-water bath. When no solvent exchange in the succeeding growth process was involved, the as-obtained Ru$_{hcp}$@Ru$_{hcp}$ and Ru$_{hcp}$@Ru$_{fcc}$ nanocrystals (suspended in EG and TEG, respectively) were directly used without any collection or washing. If a different solvent was involved in the following steps, the products were collected by precipitation with acetone (acetone/EG = 3:1 v/v) and washed three times with a mixture of acetone and ethanol (3:1 v/v) under centrifugation before redispersion in EG or TEG (*ca.* 1.41 mg/mL) for further use.

Synthesis of Ru_{hcp}@Ru_{hcp}@Ru_{hcp}, Ru_{hcp}@Ru_{hcp}@Ru_{fcc}, Ru_{hcp}@Ru_{fcc}@Ru_{hcp}, and Ru_{hcp}@Ru_{fcc}@Ru_{fcc} Nanocrystals. For the second round of overgrowth, a protocol similar to what was used for the first ground was repeated (1 mL of Ru_{hcp}@Ru_{hcp} or Ru_{hcp}@Ru_{fcc} seeds, temperature of 180°C, injection rate of 0.5 mL h^{-1}, and reaction time of 22 h), except that the Ru(III) precursor solution containing 5 mg of $Ru(acac)_3$ and 25 mg of PVP was replaced by a mixture of 2.5 mg of $Ru(acac)_3$ and 50 mg of PVP. EG was used for both the precursor and growth solutions to deposit *hcp*-Ru shell onto Ru_{hcp}@Ru_{hcp} or Ru_{hcp}@Ru_{fcc} seeds, while the formation of *fcc*-Ru shell requires the use of TEG. A summary of the experimental details can be found in Table 1.

Table 1. Summary of reaction conditions for the synthesis of Ru nanocrystals with tunable sizes and crystal phases.

**Sample**	**Ru(III) precursor solution**			**Ru seed suspension**			**Type of solvent***
	$Ru(acac)_3$ (mg)	Solvent (mL)	PVP (mg)	Seed (mg/mL)	Seed (mL)	PVP (mg)	
Ru_{hcp}@Ru_{hcp}	5	10	50	1.35	1	25	EG
Ru_{hcp}@Ru_{fcc}	5	10	50	1.35	1	25	TEG
Ru_{hcp}@Ru_{hcp}@Ru_{hcp}	2.5	10	50	1.41	1	50	EG
Ru_{hcp}@Ru_{hcp}@Ru_{fcc}	2.5	10	50	1.41	1	50	TEG
Ru_{hcp}@Ru_{fcc}@Ru_{hcp}	2.5	10	50	1.41	1	50	EG
Ru_{hcp}@Ru_{fcc}@Ru_{fcc}	2.5	10	50	1.41	1	50	TEG

*All syntheses in the first and second rounds of overgrowth maintained a constant temperature (180 °C), injection rate (0.5 mL h^{-1}), and reaction duration (22 h). The same polyol (EG or TEG) was applied to the preparation of both the precursor solution and the seed suspension.

Quantitative Analysis of Reduction Kinetics. In a typical study, 0.1 mL aliquot was sampled from the reaction solution at different time points of a synthesis and quenched in the ice bath to prevent further reaction. The aliquot was then mixed with 9.9 mL of acetone to help precipitate out all the particles followed by centrifugation to only leave behind the unreacted Ru(III) precursor in the supernatant. The supernatant was then collected and diluted for elemental analysis.

Characterizations. Transmission electron microscopy (TEM) images were taken on a Hitachi HT7700 microscope operated at 120 kV. The sample for TEM analysis was prepared by drop-

casting an ethanol suspension of the nanocrystals on a carbon-coated copper grid, followed by drying under ambient conditions. High-resolution transmission electron microscopy (HRTEM) images were obtained using an FEI Tecnai G2 F30 TEM operated at 300 kV. X-ray diffraction (XRD) patterns were recorded on a PANalytical X'Pert PRO Alpha-1 diffractometer using a 1.8 kW ceramic copper tube source. An inductively-coupled plasma mass spectrometer (ICP-MS, Perkin Elmer, NexION 300Q) was used to determine the content of Ru in the sample.

DFT calculations. All DFT calculations were performed using the Vienna *Ab initio* Simulation Package (VASP)[2-4] with the Projector Augmented Wave (PAW) method.[5] Each parameter (*i.e., k* points, energy cutoff, vacuum spacing) was chosen after convergence tests with respect to the surface and total energy of the DFT calculations (Figure S6). A plane wave energy cutoff of 450 eV was used with the Perdew-Burke-Ernzerhof (PBE) parameterization of the Generalized Gradient Approximation (GGA) exchange-correlation functional[6] with 10^{-5} eV as a criterion for energy convergence. For the sampling of the first Brillouin zone, Monkhorst-Pack grids were used.[7] All the atomic positions were optimized until their corresponding forces were smaller than 0.05 eV/Å. To include long-range van der Waals (vdW) interactions, the DFT-D3 method with the Becke-Jonson (BJ) damping function was used.[8]

Ru(0001) was used as a representative facet of *hcp*-Ru, as it is thermodynamically the most stable facet of Ru.[9] For Ru(0001), a $(6 \times 6 \times 1)$ *k*-point mesh was used for a hexagonal $c(2 \times 2)$ unit cell that was four layers thick. Our calculations gave optimized lattice constants of a = b = 2.71 Å, c = 4.27 Å. The top two layers were relaxed with adsorbed species, while the bottom two layers were fixed at the bulk positions. The dipole correction was employed in the *z*-direction normal to the slab when there were adsorbed species (*i.e.*, solvent, Ru_NO_M with M adsorbed O and N adsorbed Ru) on the slab surface, to avoid unphysical interactions between periodic cells. All slabs included a vacuum spacing of 13 Å in the *z*-direction. For the energies of single solvent molecules (*i.e.*, TEG or EG), a cubic unit cell with a side length of 20 Å and the Γ point were used for geometry optimization in the gas phase.

$\Delta E_{Ru_NO_M}$ will be used to indicate the relative stabilities of Ru atoms with *fcc* or *hcp* packing in our DFT models. $\Delta E_{Ru_NO_M}$ is defined as

$$\Delta E_{Ru_NO_M} = \frac{E_{Ru(0001)+MO+NRu_{fcc}} - E_{Ru(0001)+MO+NRu_{hcp}}}{N} \quad , \qquad \text{(S1)}$$

where $E_{Ru(0001)+MO+NRu_{fcc}}$ is the total energy of an optimized Ru(0001) slab with M adsorbed O and N adsorbed Ru atoms initially placed on *fcc* sites and $E_{Ru(0001)+MO+NRu_{hcp}}$ is the total energy of a slab with the same size and same initial configuration and numbers of Ru and O atoms *except* that the Ru atoms are initially placed on *hcp* sites. It should be noted that $\Delta E_{Ru_N O_M}$ is the relative binding energy difference per Ru atom, where a negative value indicates that Ru adsorption is preferred on *fcc* sites and *hcp* Ru adsorption is preferred when $\Delta E_{Ru_N O_M}$ is positive.

AIMD Simulations. AIMD simulations were performed using VASP with the PAW method. A plane-wave energy cutoff of 450 eV was used with the PBE parameterization of the GGA exchange-correlation functional. The total energy was evaluated with an accuracy of 10^{-5} eV. For sampling of the first Brillouin zone, a single ***k*** point was used. The AIMD simulation was conducted in the NVT ensemble using the Andersen thermostat to maintain constant temperature conditions,[10] with a time step of 0.25 fs for all simulations. Grimme's DFT-D3 method with Becke-Jonson damping was used for long-range vdW interactions. We created a simulation box with a density of 1.11 g/cm^3 and 1.03 g/cm^3 for EG and TEG, respectively, to realize the density for each solvent at room temperature (*i.e.*, ρ (EG) = 1.11 g/cm^3, ρ (TEG) = 1.1 g/cm^3)[11-13]. A cubic periodic box with a side length of 15.08 Å, and a single ***k*** point was used for EG solvent. For TEG solvent, we used a cubic periodic box with a side length of *a* = *b* = 11.00 Å, *c* = 12.00 Å, and a $(2 \times 2 \times 2)$ *k*-point mesh. We optimized an initial configuration of solvent with 1 RuO_2 in a periodic box and ran an AIMD simulation with an Andersen thermostat frequency of 0.1. An initial temperature of 500 K was used to realize the experimental temperature at ~180 °C for 8 ps. Afterwards, we also ran the simulation using different temperatures (*i.e.,* 800 K, 1000 K, 1200 K, 1500 K, 2000K) to accelerate sampling of the configurations for 5 ps, 7 ps, 4.7 ps, 8ps, 8.32 ps respectively, at each temperature. We chose 2000 K to produce a desirable result, as we observed pyrolysis of EG (or TEG) at a temperature of 2500 K.

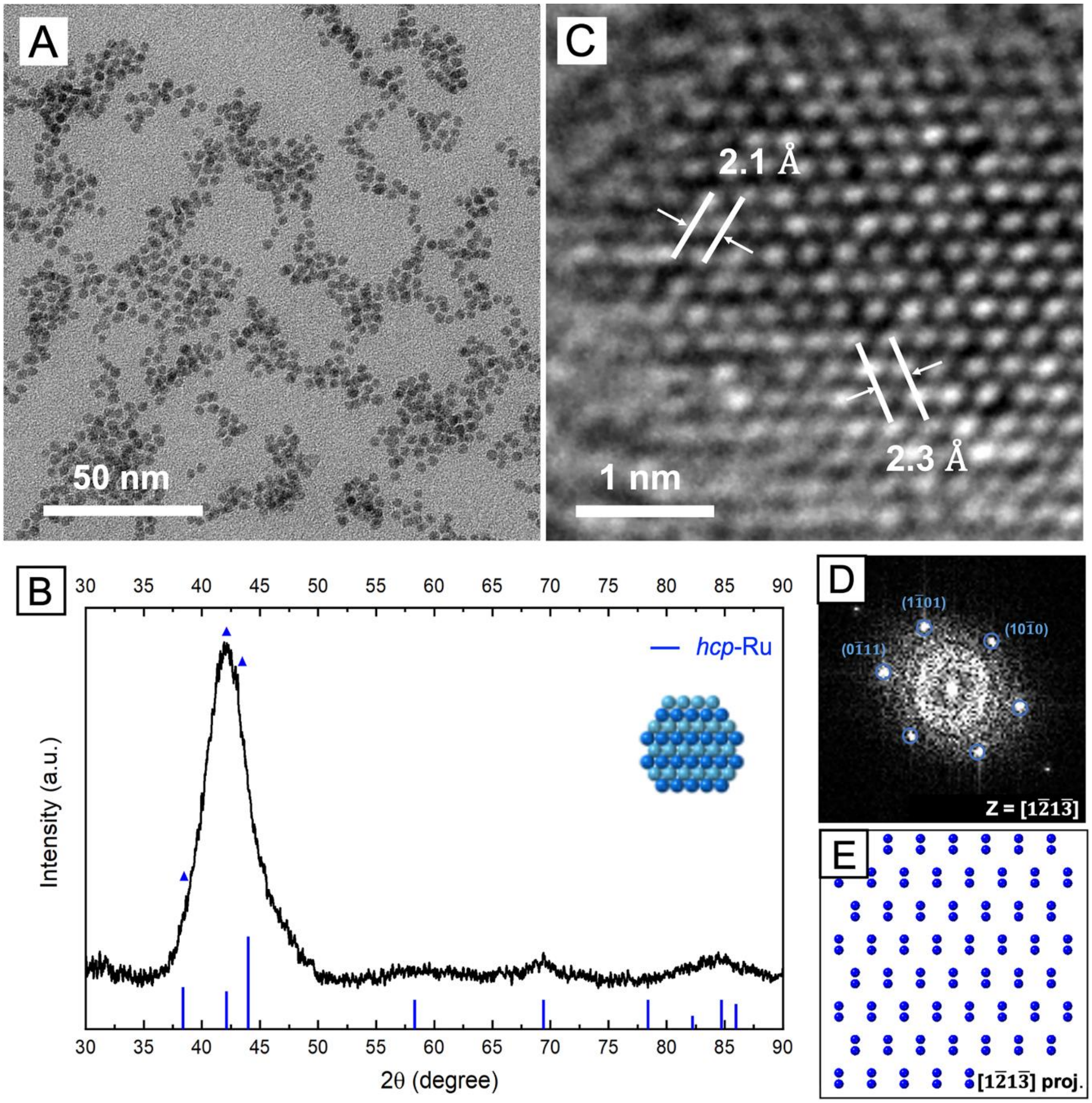

Figure S1. Structural characterizations of the 3.1 nm Ru_{hcp} nanocrystals serving as seeds for the first round of overgrowth. (A) TEM image. (B) XRD pattern of the nanocrystals, confirming their *hcp* crystal structure. The blue lines correspond to the characteristic peaks of *hcp*-Ru (JCPDS No. 06-0663). The inset is an atomic model of the cross-section of the Ru_{hcp} nanocrystal. (C) HRTEM image of an individual nanocrystal and (D) the corresponding FFT pattern. (E) Projected atomic model of the *hcp*-Ru lattice viewed along the [1$\bar{2}$1$\bar{3}$] direction.

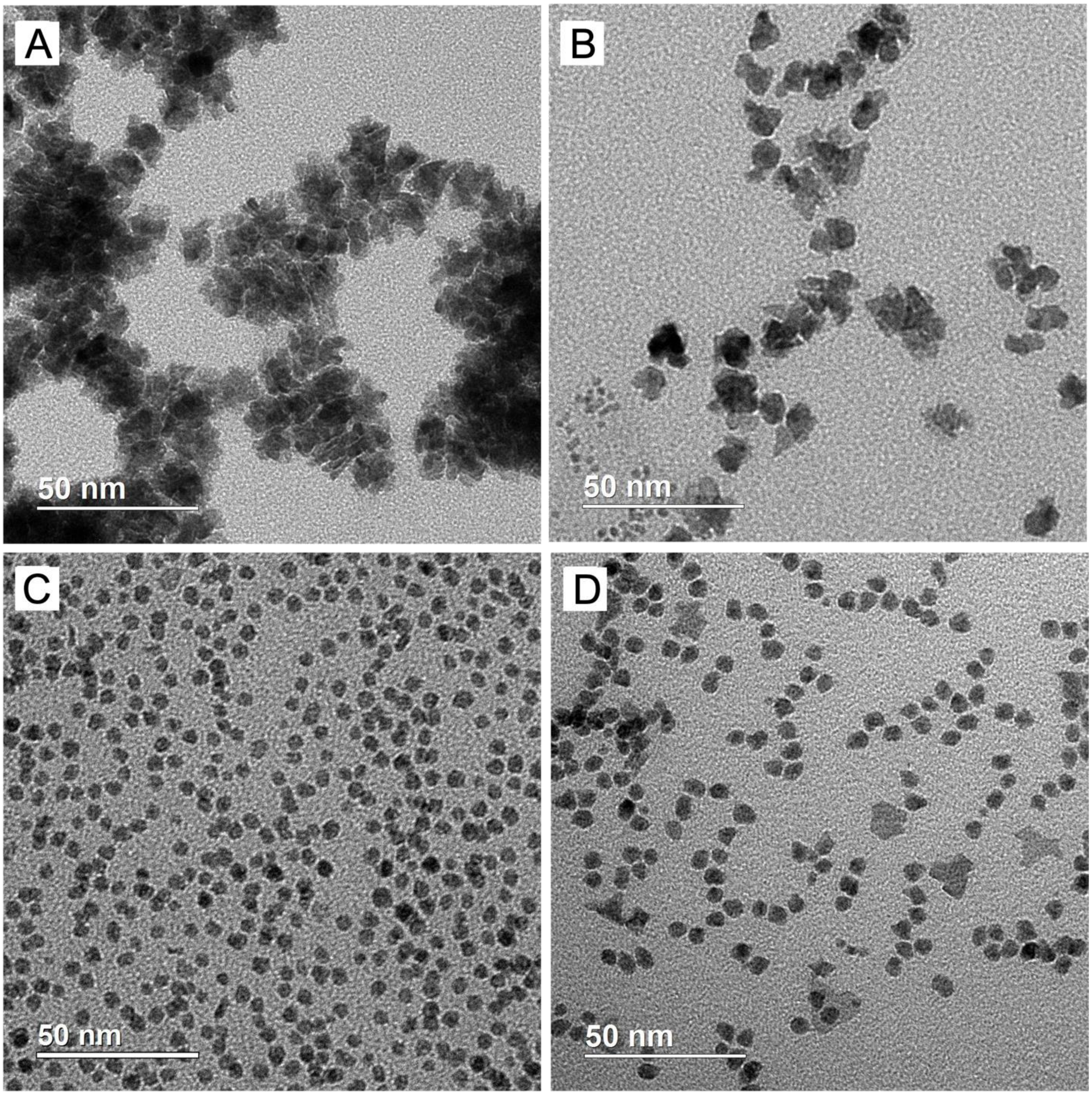

Figure S2. TEM images of the Ru@Ru nanocrystals derived from the first round of overgrowth by following the standard protocol, except for (A) the use of an injection rate of 4 mL h^{-1}, (B, C) the change of reaction temperature to (B) 200 °C and (C) 160 °C, and (D) the use of $RuCl_3$ as the precursor.

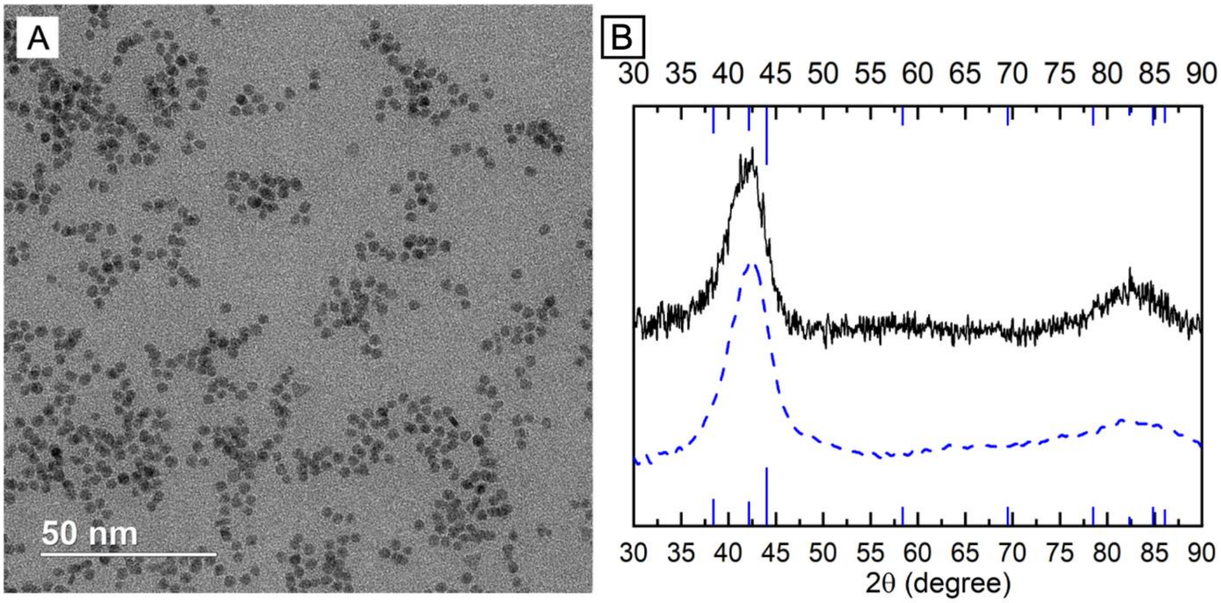

Figure S3. (A) TEM image and (B) XRD pattern of the 3.1 nm Ru_{hcp} seeds after being mixed with PVP in TEG and heated at 180 °C for 22 h without introducing the precursor (black trace) and that of the freshly prepared sample (blue dashed trace). The blue bars at the bottom and top correspond to the characteristic peaks of *hcp*-Ru (JCPDS No. 06-0663).

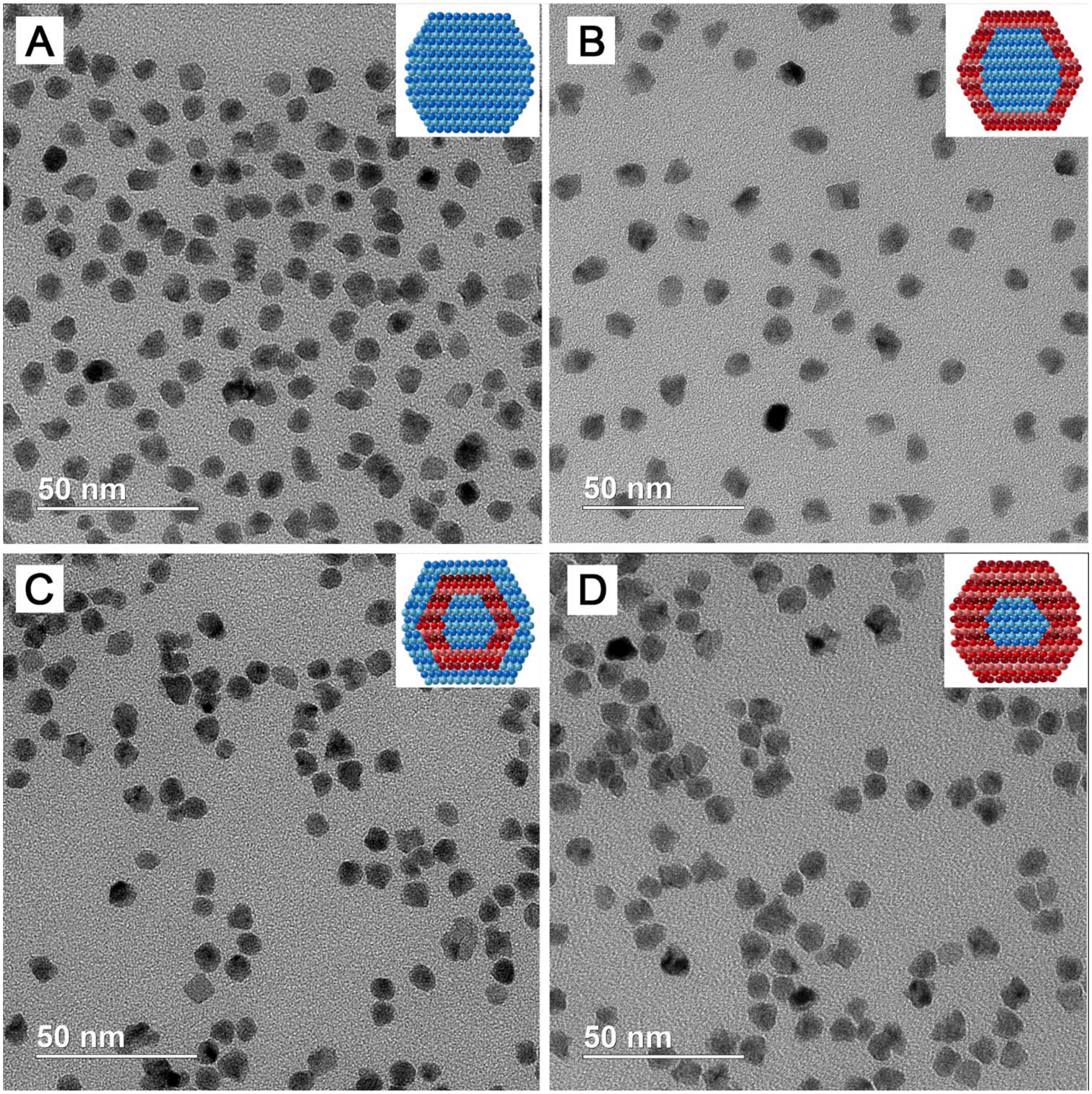

Figure S4. Samples of the 7 nm Ru nanocrystals with an *hcp* or *fcc* phase in the outermost shell obtained from the second round of overgrowth. (A, B) TEM images of the Ru_{hcp}@Ru_{hcp}@Ru_{hcp} and Ru_{hcp}@Ru_{hcp}@Ru_{fcc} nanocrystals synthesized from the Ru_{hcp}@Ru_{hcp} seeds using (A) EG and (B) TEG, respectively. (C, D) TEM images of the Ru_{hcp}@Ru_{fcc}@Ru_{hcp} and Ru_{hcp}@Ru_{fcc}@Ru_{fcc} nanocrystals synthesized from the Ru_{hcp}@Ru_{fcc} seeds using (C) EG and (D) TEG, respectively. The insets are the corresponding atomic models in a cross-sectional view.

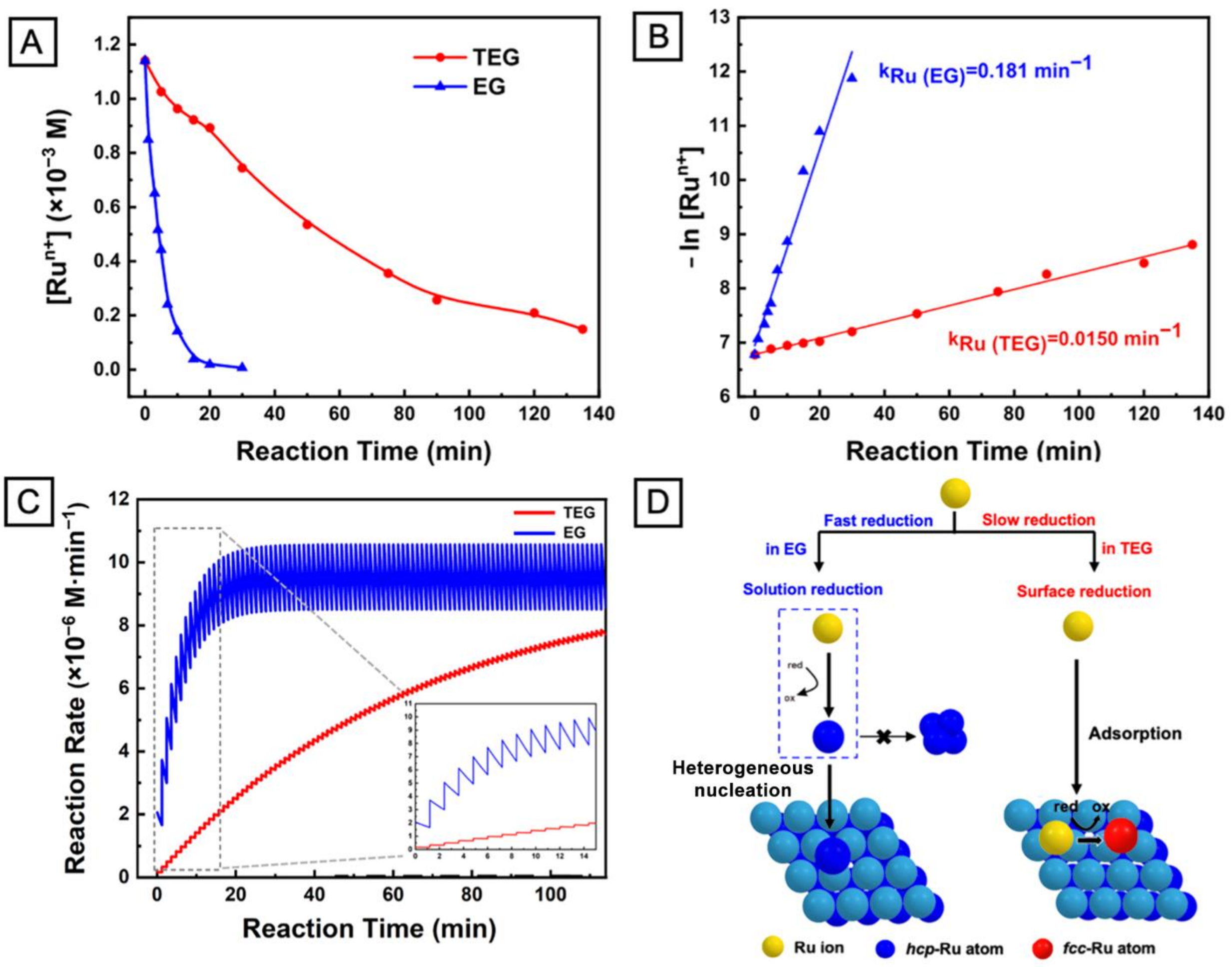

Figure S5. Quantitative analysis of the reduction kinetics of $Ru(acac)_3$ precursor in EG and TEG as mediated by the 3.1 nm Ru_{hcp} seeds. (A) Plots of the concentrations of remaining precursor in the reaction solutions as a function of time for one-shot injection involving EG (red curve) and TEG (blue curve), respectively. (B) Plots showing the linear relationship between −ln([precursor]) and reaction time and the fittings based on the pseudo-first-order kinetics. (C) Simulated reaction rates as a function of reaction time in the case of dropwise titration at 0.5 mL h^{-1} for the two systems. The inset shows a segment of the curves in the first 15 min of the synthesis. (D) Schematic of the reduction kinetics and pathways that are supposed to be taken by the precursor in EG and TEG for the formation of *hcp*-Ru and *fcc*-Ru overlayers, respectively.

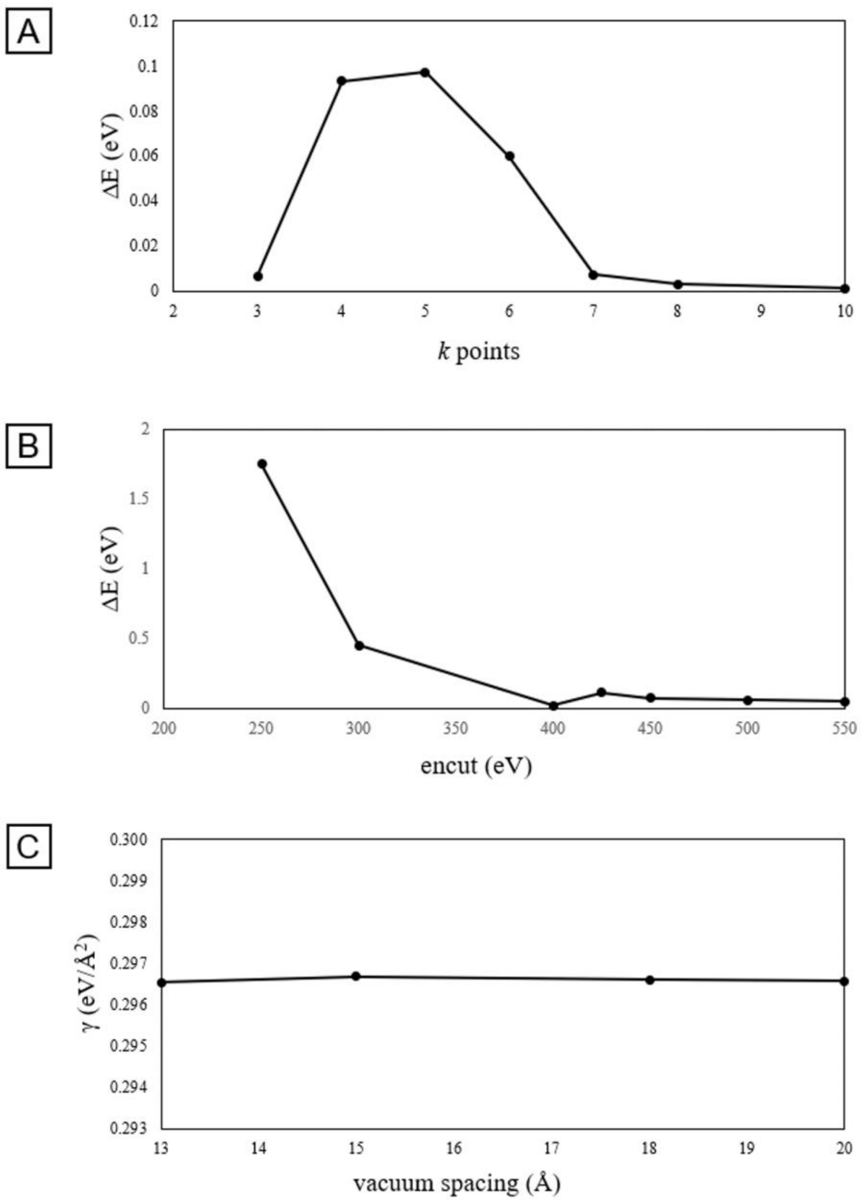

Figure S6. Convergence tests for the single-point energies of four-layer Ru(0001) slabs. The number of *k* points, value of the cut-off energy, and vacuum spacing were chosen to achieve an energy tolerance of 0.01 eV. In (A), we show the convergence of the calculations regarding *k* points – with $(N \times N \times 1)$ meshes, where N is the value on the x axis. The y axis value ΔE at N is the energy difference between point N and point N-1. In (B), we show the convergence of the energy cut-off (encut) using ΔE, where ΔE has the same definition as in (A). In (C) we show the value of the surface energy (γ) as a function of vacuum spacing (Å). The surface energy was calculated using:

$$\gamma_{\mathrm{Ru}} = \frac{E_{\mathrm{Ru}} - N_{\mathrm{Ru}} E_{\mathrm{Ru}}^{\mathrm{Bulk}}}{A_{\mathrm{surf}}} - \gamma_{\mathrm{Ru}}^{\mathrm{fixed}} , \tag{S2}$$

where E_{Ru} is the energy of the Ru slab, N_{Ru} is the number of Ru atoms, $E_{\mathrm{Ru}}^{\mathrm{Bulk}}$ is the DFT bulk energy per Ru atom, A_{surf} is the surface area of the Ru slab. Since only one side of the Ru slab is optimized in our calculations, we subtracted $\gamma_{\mathrm{Ru}}^{\mathrm{fixed}}$ – the surface energy of a bare Ru surface slab with atoms fixed at the bulk coordinates.

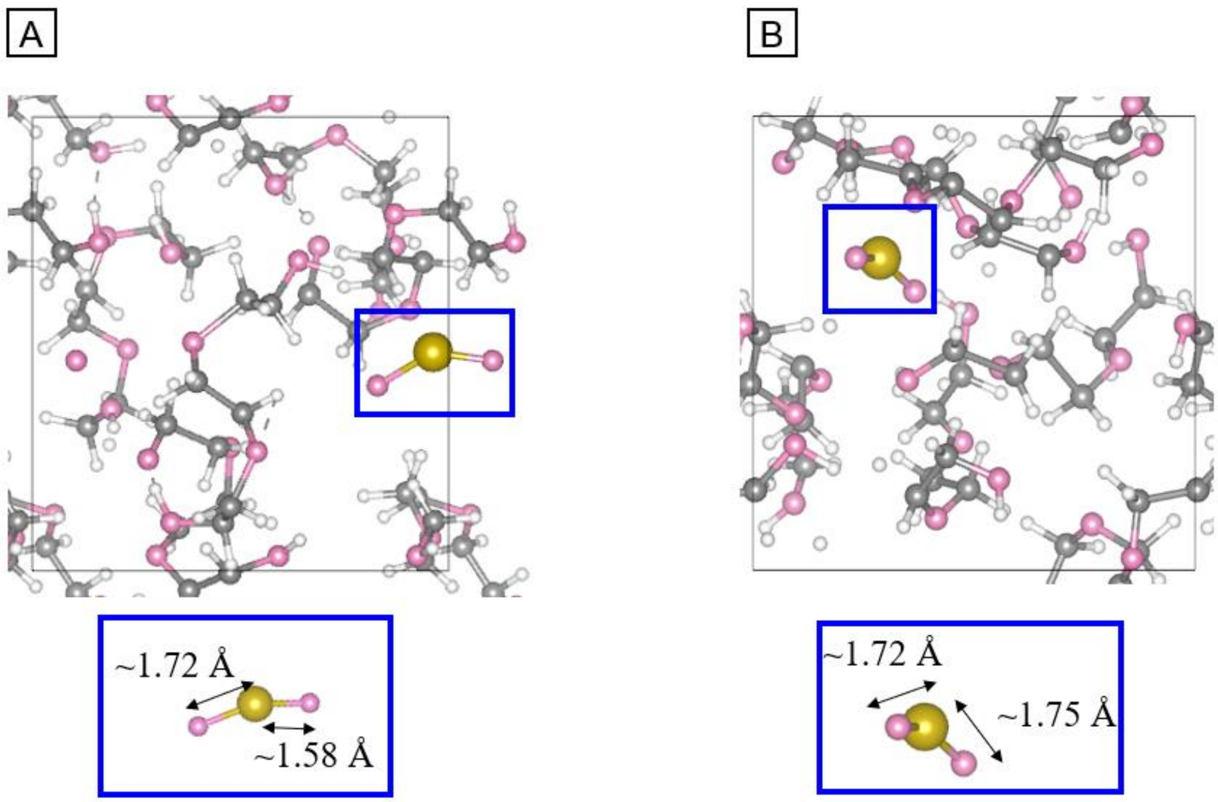

Figure S7. AIMD result of one RuO_2 in the TEG solution at ~2000 K. Different initial placement of RuO_2 molecule at (A) the end of TEG molecule and (B) in the middle of TEG molecule. Yellow: Ru in the solution; pink: oxygen; grey: carbon; white: hydrogen.

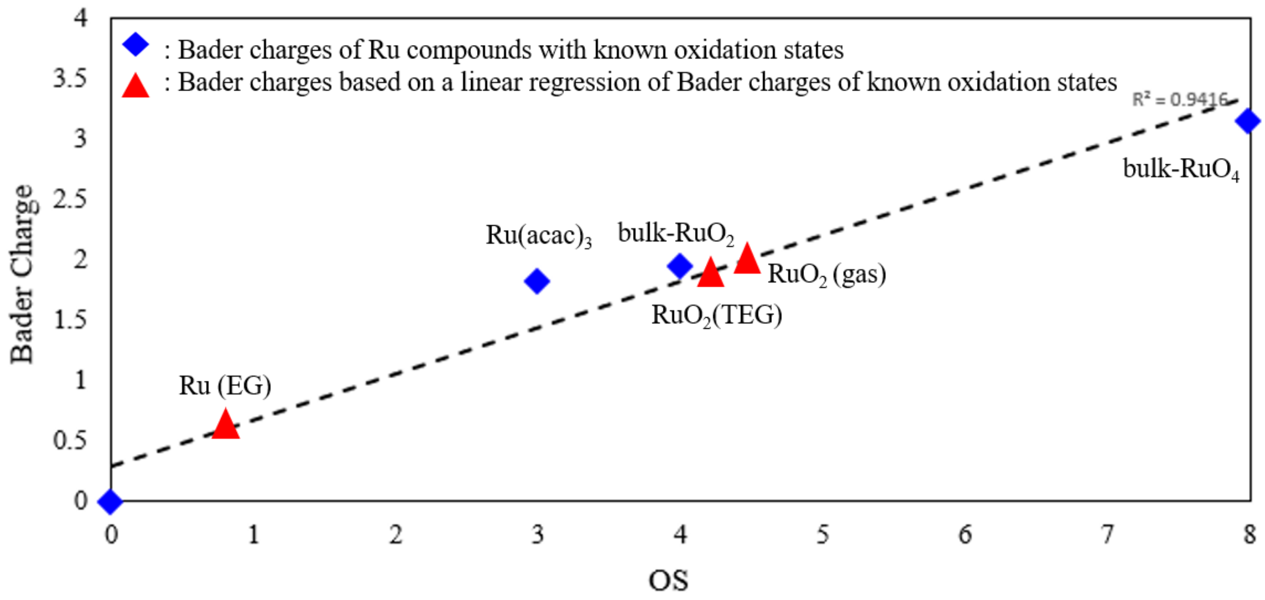

	Bader charge (Net atomic charge of Ru)	OS
Ru atom	0	0
$Ru(acac)_3$	1.83	3
bulk-RuO_2	1.95	4
bulk-RuO_4	3.15	8
RuO_2(TEG)	1.87	4.12
RuO_2(gas)	1.99	4.43
Ru (EG)	0.68	0.98

Figure S8. Calculated Bader charge *versus* oxidation state (OS) using linear regression for Ru compounds based on known oxidation states. The OS and Bader charge of RuO_2 (TEG) is the average OS and Bader charge of RuO_2 in (Figure S7A) and (Figure S7B). Ru (EG) indicates the Bader charge and the OS of the Ru atom in EG in Figure 6D.

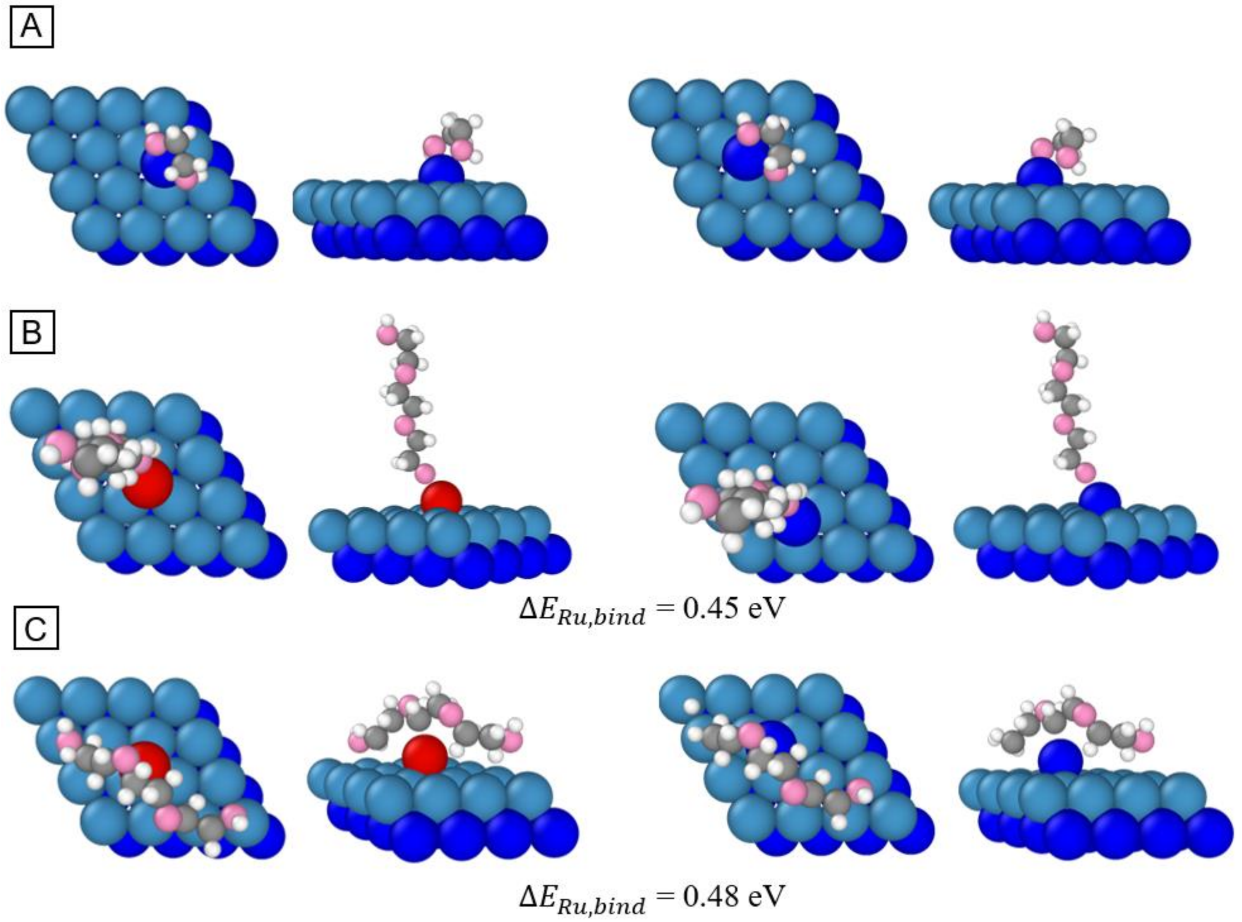

Figure S9. Top and side view of Ru adatom at *fcc* and *hcp* site in EG (or TEG) on *hcp*-Ru (0001). (A) Initial placements of Ru adatom at *fcc* and *hcp* on *hcp*-Ru (0001) were optimized to *hcp* sites. (B, C) The relative binding energy of the Ru adatom was calculated using:

$$\Delta E_{Ru,bind} = E_{Ru(0001)+EG\ (or\ TEG)+Ru_{fcc}} - E_{Ru(0001)+EG\ (or\ TEG)+Ru_{hcp}}, \qquad \text{(S3)}$$

where $E_{Ru(0001)+EG\ (or\ TEG)+Ru_{fcc}}$ is the total energy of an optimized Ru(0001) slab with solvent and an adsorbed Ru atom initially placed on *fcc* site and $E_{Ru(0001)+EG\ (or\ TEG)+Ru_{hcp}}$ is the total energy of an optimized Ru(0001) slab with solvent and an adsorbed Ru atom initially placed on *hcp* site. Negative $\Delta E_{Ru,bind}$ indicates *hcp*-Ru adatom is preferred whereas a positive value indicates *fcc*-Ru adatom is preferred. Sky, blue: *hcp*-Ru template; pink: oxygen; red: *fcc*-Ru; white: hydrogen; grey: carbon.

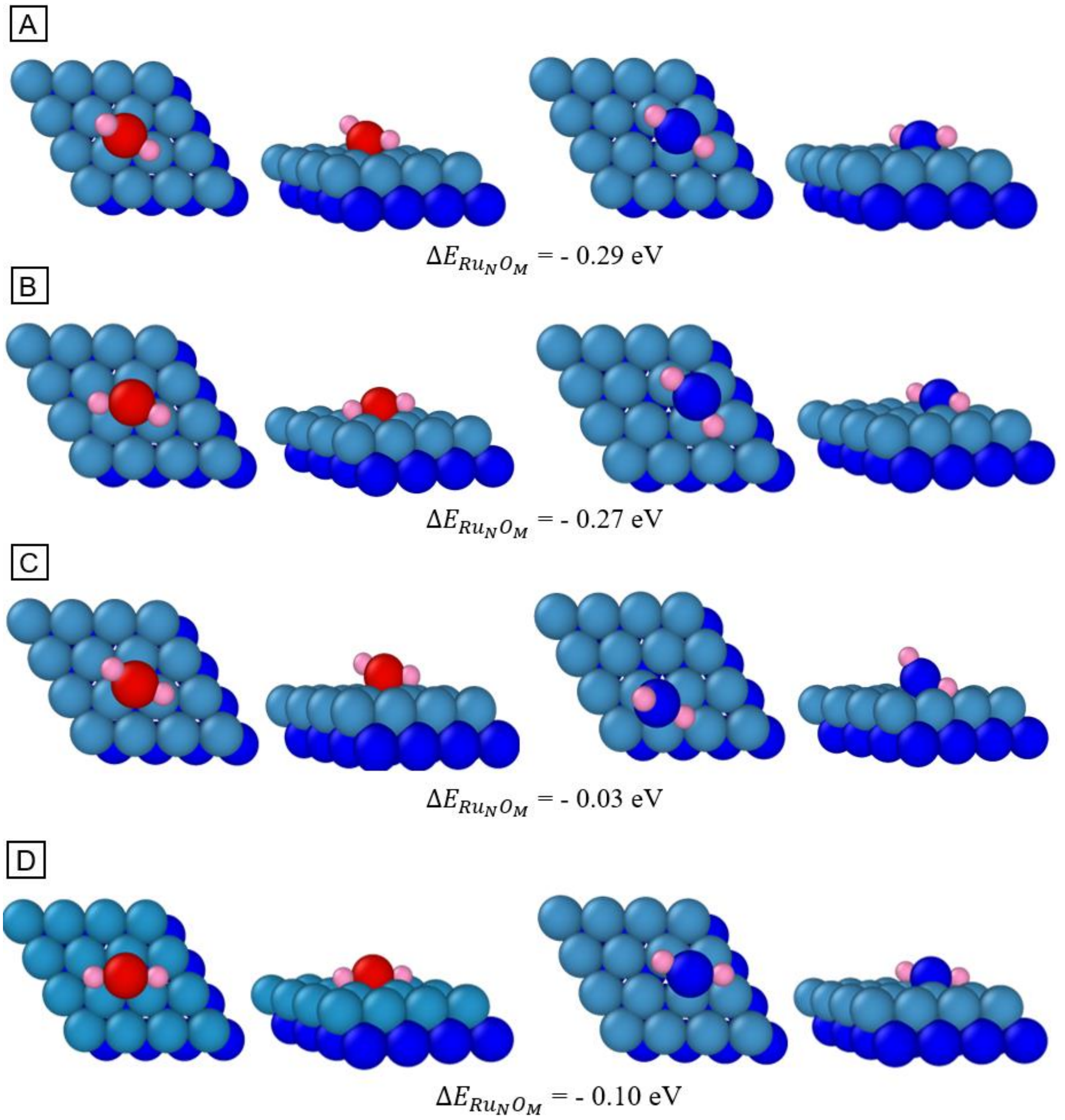

Figure S10. Top and side view of one RuO_2 at *fcc* and *hcp* site on *hcp*-Ru(0001). The relative binding energy of RuO_2, given by Equation (S1) in the experimental section, is indicated. Sky and blue: *hcp*-Ru; red: *fcc*-Ru; pink: oxygen.

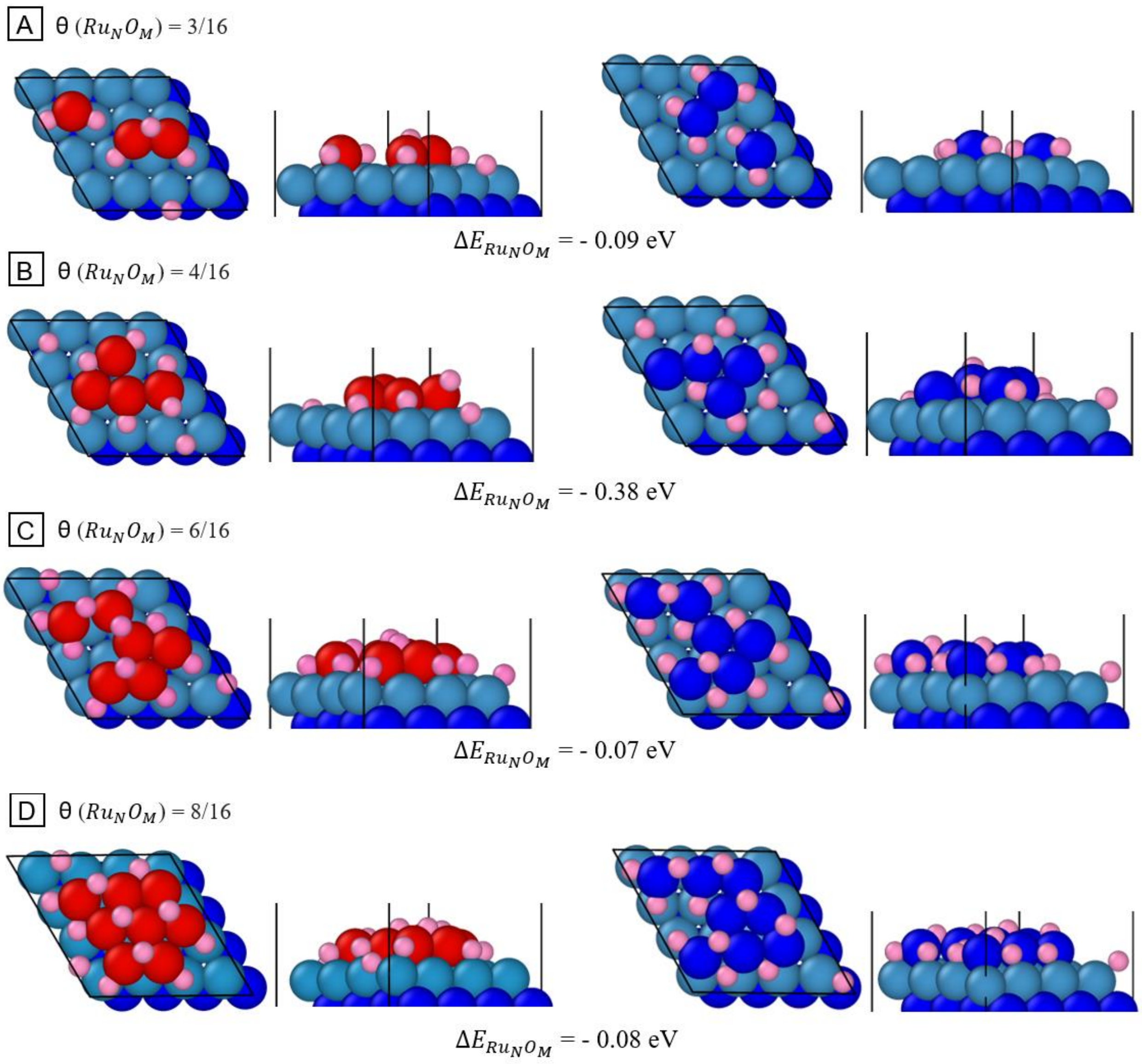

Figure S11. Top and side view of Ru_NO_M adsorption on the *hcp*-Ru (0001) slab models with different coverages of Ru_NO_M (θ). The relative energy of the slabs per Ru atom, given by Equation (S1) in the experimental section, is indicated. Sky, blue: *hcp*-Ru seed; red: *fcc*-Ru overlayer; pink: oxygen.

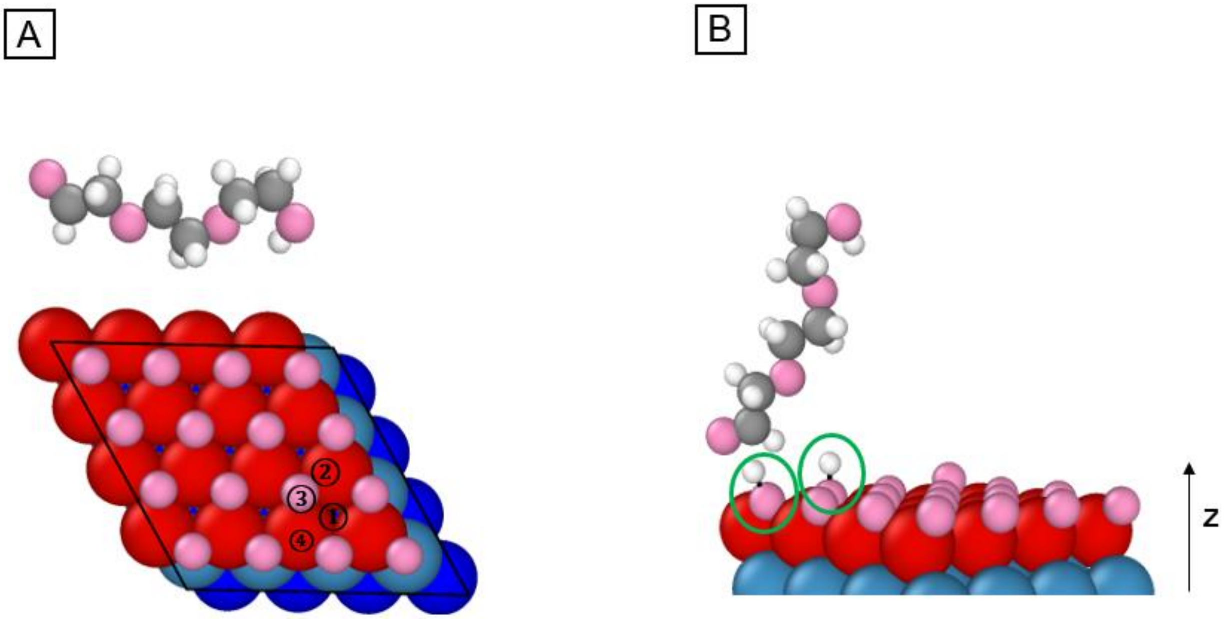

Figure S12. (A) Adsorption of the hydroxyl group in TEG on the *fcc*-Ru_NO_M at 1) hollow-*hcp* site, 2) Ru atom at *fcc* site closer to nearby *hcp* oxygen atom, 3) on top of *hcp* oxygen atom in Ru_NO_M, and 4) on top of *fcc*-Ru atom in Ru_NO_M. (B) Snapshot of final configuration after creating hydroxyl group assisted by TEG. Sky and blue: *hcp*-Ru; red: *fcc*-Ru; pink: oxygen; grey: carbon; white: hydrogen.

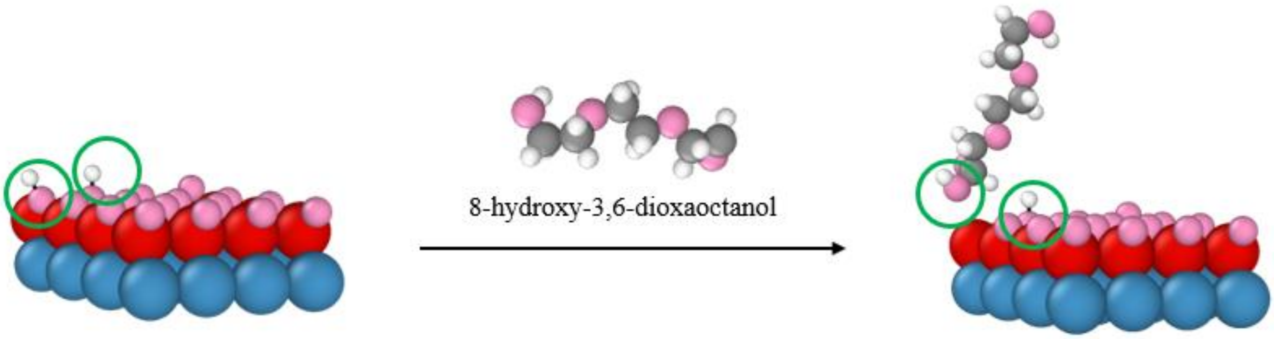

Figure S13. Removal of hydroxyl group from the surface by the following oxidized TEG solvent (*i.e.*, 8-hydroxy-3,6-dioxaoctanol). Green circle indicates the created hydroxyl group after hydrogen atom leaving the solvent. Sky, blue: *hcp*-Ru seed; red: *fcc*-Ru overlayer; pink: oxygen; grey: carbon, white: hydrogen.

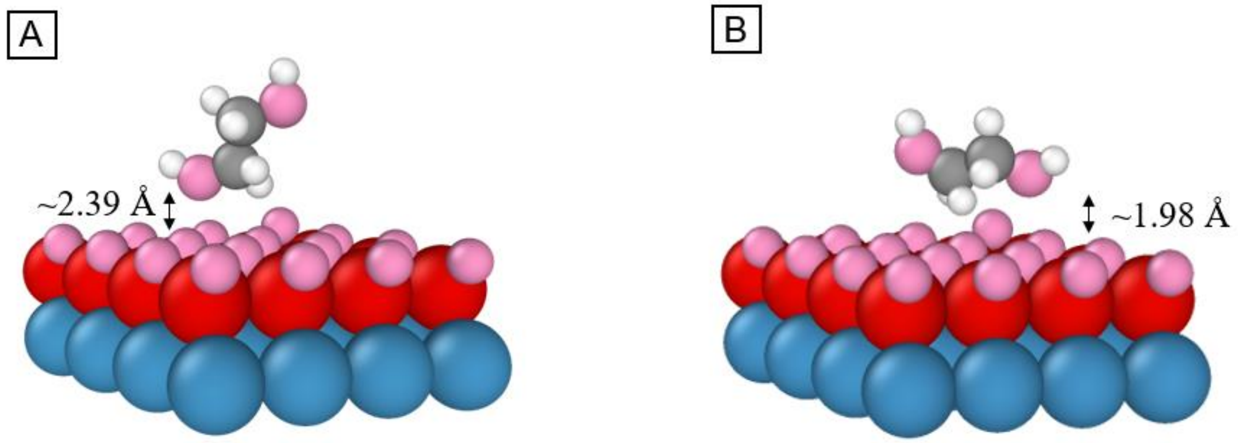

	E_{bind} (eV)	$E_{electronic}$ (eV)	E_{vdW} (eV)
A	0.25	0.04	0.21
B	0.30	0.02	0.28

Figure S14. Optimized configurations after EG molecule placements on the *fcc*-Ru_NO_M covering *hcp*-Ru (0001) and its corresponding binding energy (E_{bind}) calculated using:

$$E_{bind} = E_{Ru_NO_M} + E_{EG} - E_{EG-Ru_NO_M} \quad (S4)$$

$$E_{bind} = E_{electronic} + E_{vdW} \quad (S5)$$

where $E_{Ru_NO_M}$ is the optimized total energy for a *hcp*-Ru (0001) slab with an *fcc*-Ru_NO_M overlayer. E_{EG} is the energy of EG molecule in the gas phase. $E_{EG-RuNOM}$ is the computed energy of a system where EG is placed on the *fcc*-Ru_NO_M overlayer. E_{bind} was decomposed to two contributions, $E_{electronic}$ and E_{vdW}. To obtain $E_{electronic}$, we froze each system in Equation (S4) after geometry optimization and ran a single point energy calculation by excluding vdW term. Sky: *hcp*-Ru template; pink: oxygen; red: *fcc*-Ru overlayer; white: hydrogen; grey: carbon.

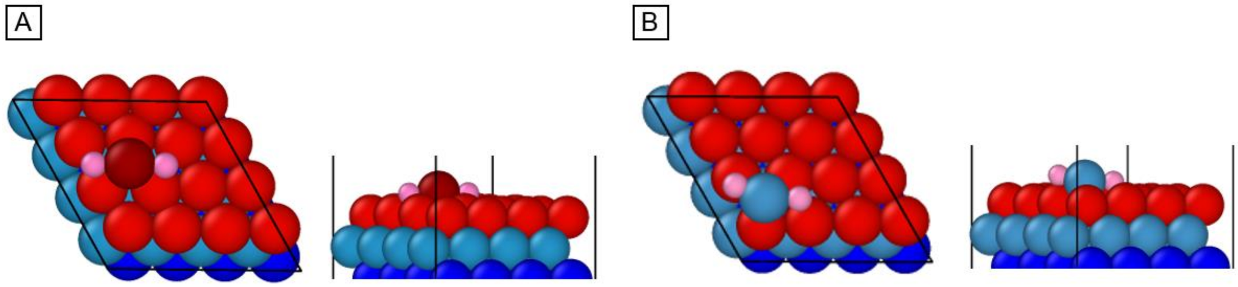

$\Delta E_{Ru_N O_M}$ = - 0.03 eV

Figure S15. Top and side view of the second RuO_2 overlayer at (A) *fcc* and (B) *hcp* sites on the *hcp*-Ru template. The relative energy of the slabs per Ru atom, given by Equation (S1) in the experimental section, is indicated. Sky and blue: *hcp*-Ru seed; red and brown: *fcc*-Ru; pink: oxygen.

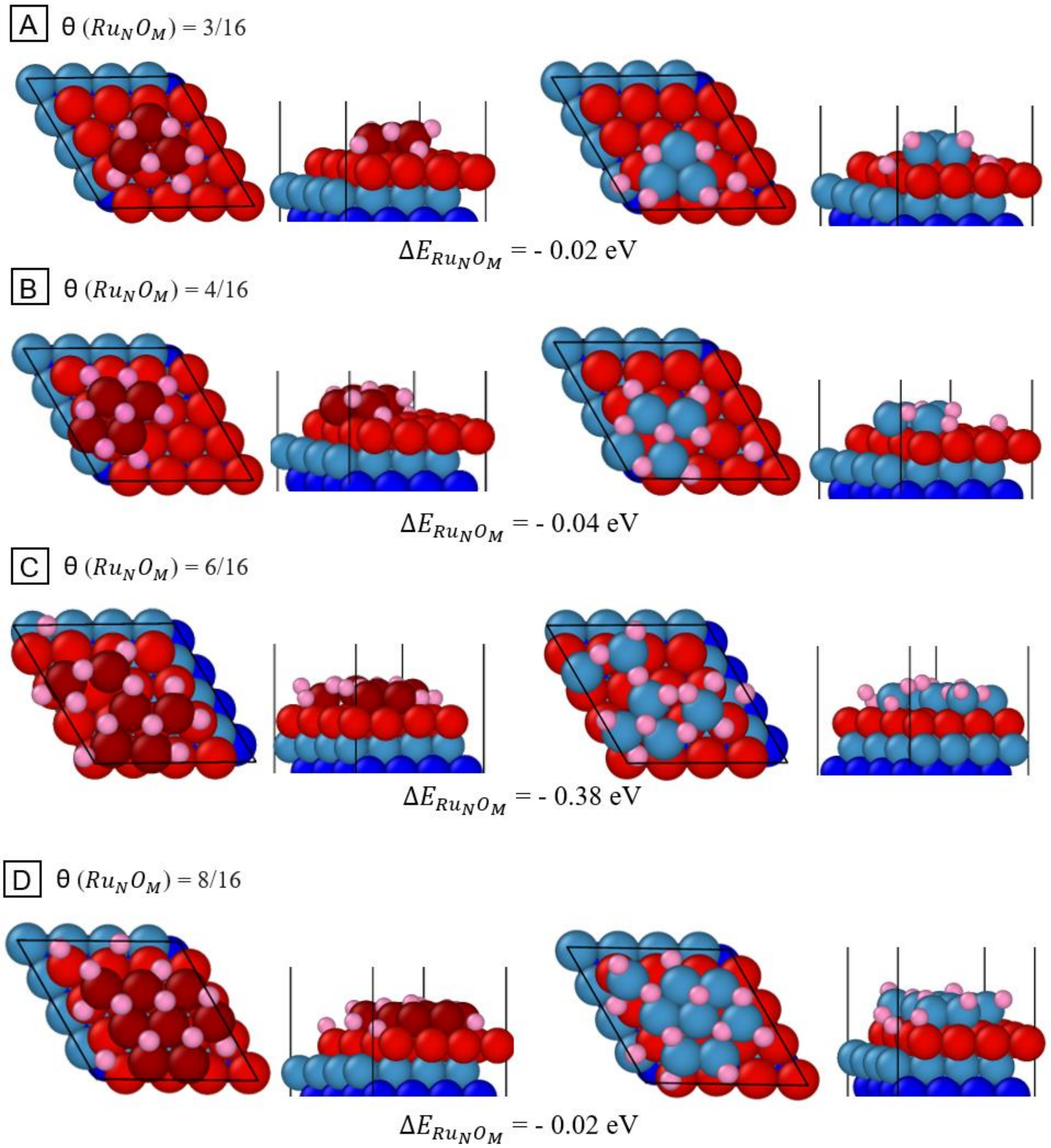

Figure S16. Top and side view of the second Ru_NO_M overlayer on the Ru (0001) slab models with different coverages of Ru_NO_M (θ). The relative energy of the slabs per Ru atom, given by Equation (S1) in the experimental section, is indicated. Sky, blue: *hcp*-Ru seed; red, brown: *fcc*-Ru overlayer; pink: oxygen.

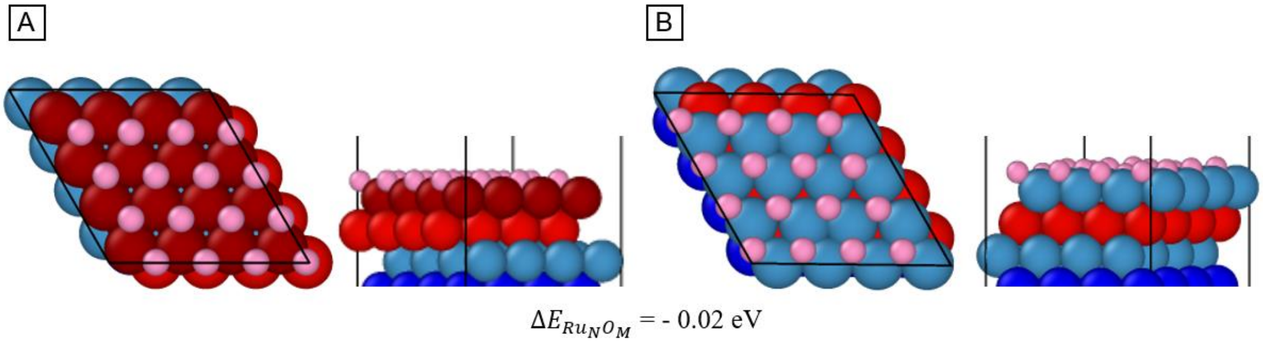

Figure S17. Top and side view of the second Ru_NO_M overlayer at (A) *fcc* and (B) *hcp* on the *hcp*-Ru template. The relative energy of the slabs per Ru atom, given by Equation (S1) in the experimental section, is indicated. Sky and blue: *hcp*-Ru seed; red and brown: *fcc*-Ru overlayer; pink: oxygen.

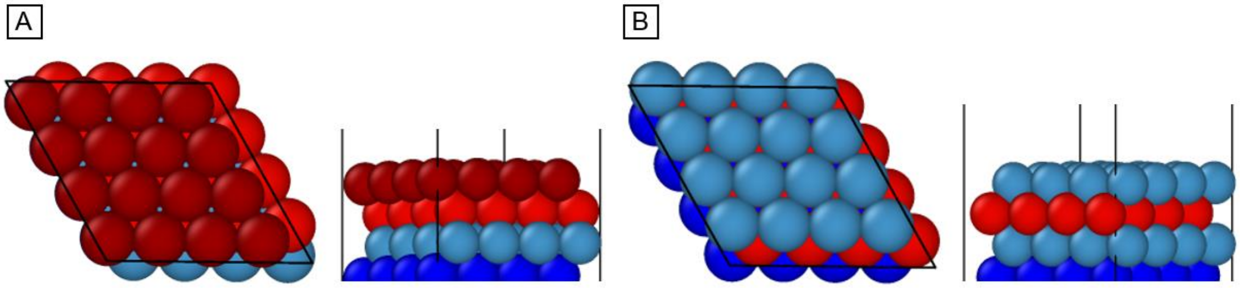

$\Delta E_{Ru_N O_M} = 0.19\ \text{eV}$

Figure S18. Top and side view of the second Ru overlayer at (A) *fcc* and (B) *hcp* on the *hcp*-Ru template. The relative energy of the slabs per Ru atom, given by Equation (S1) in the experimental section, is indicated. Sky, blue: *hcp*-Ru seed; red, brown: *fcc*-Ru overlayer.

REFERENCES

(1) Kusada, K.; Kobayashi, H.; Yamamoto, T.; Matsumura, S.; Sumi, N.; Sato, K.; Nagaoka, K.; Kubota, Y.; Kitagawa, H. Discovery of face-centered-cubic ruthenium nanoparticles: facile size-controlled synthesis using the chemical reduction method. *J. Am. Chem. Soc.* **2013**, *135*, 5493–5496.

(2) Kresse, G.; Hafner, J. Ab Initio molecular dynamics for liquid metals. *Phys. Rev. B* **1993**, *47*, 558–561.

(3) Kresse, G.; Furthmüller, J. Efficient iterative schemes for Ab Initio total-energy calculations using a plane-wave basis set. *Phys. Rev. B* **1996**, *54*, 11169–11186.

(4) Kresse, G.; Hafner, J. Ab Initio molecular-dynamics simulation of the liquid-metalamorphous-semiconductor transition in germanium. *Phys. Rev. B* **1994**, *49*, 14251–14269.

(5) Blöchl, P. E. Projector augmented-wave method. *Phys. Rev. B* **1994**, *50*, 17953–17979.

(6) Perdew, J. P., Burke, K. & Ernzerhof, M. Generalized gradient approximation made simple. *Phys. Rev. Lett.* **1996**, *77*, 3865–3868.

(7) Monkhorst, H. J.; Pack, J. D. Special points for brillouin-zone integrations. *Phys. Rev. B* **1976**, *13*, 5188–5192.

(8) Grimme, S., Antony, J., Ehrlich, S. & Krieg, H. A consistent and accurate ab initio parametrization of density functional dispersion correction (DFT-D) for the 94 elements H-Pu. *J. Chem. Phys.* **2010**, *132*, 154104.

(9) Herron, J. A., Tonelli, S.; Mavrikakis, M. Atomic and molecular adsorption on Ru(0001). *Surf. Sci.* **2013**, *614*, 64–74.

(10) E, W.; Li, D. The Andersen thermostat in molecular dynamics. *Commun. Pure Appl. Math*. **2008**, *61*, 96-136.

(11) Holman, J.P. *Heat Transfer*, 9th ed.; McGraw-Hill Companies, 2001; pp 600-606.

(12) Incropera, F. P.; Dewitt, D. P.; Bergman, T. L.; Lavigne, A. S. *Fundamentals of heat and mass transfer*, 6th ed.; John Wiley and Sons, 2006, pp. 941–950.

(13) Carvalho, P. J.; Fonseca, C. H. G.; Moita, M. L. C. J.; Santos, A. F. S.; Coutinho, J. A. P. Thermophysical properties of glycols and glymes. *J. Chem. Eng. Data* **2015**, 60, 3721–3737.
